# Decoding the Alphabet Soup: A Practical Guide to Genetic Testing in Hyperkinetic Movement Disorders

**DOI:** 10.5334/tohm.971

**Published:** 2025-06-26

**Authors:** Claudia Del Gamba, Giulietta Maria Riboldi

**Affiliations:** 1 The Marlene and Paolo Fresco Institute for Parkinson’s and Movement Disorders, Department of Neurology, NYU Langone Health, NY, US; 2 Azienda USL Toscana Centro, Neurology Unit, Nuovo Ospedale di Prato Santo Stefano, Prato, IT

**Keywords:** next generation sequencing, repeat expansion, genotype-phenotype, variant of uncertain significance, hyperkinetic movement disorders, genetic tests

## Abstract

**Background:**

The diagnosis of genetic hyperkinetic movement disorders has become increasingly more complex as new genes are discovered and technologies offer new diagnostic possibilities. As a result, the choice of appropriate gene testing and the interpretation of the results can become difficult to navigate for movement disorder experts and clinicians. In parallel, research is becoming crucial to pair with clinical assessments in order to explore advanced sequencing technologies and allow new genes discovery.

**Methods:**

Systematic review of genetic forms of hyperkinetic movement disorders and of the most relevant genetic terminology was performed.

**Results:**

Comprehensive descriptions of genetic lexicon, testing selection, and complex genetic findings related to hyperkinetic movement disorders are reported.

**Discussion:**

Here we discuss the terminology of genetic diagnosis that is now part of the clinical practice, the difficulties related to the interpretation of complex genetic results, and provide guidance and tips for gene testing selection in order not to miss important diagnosis of genetic hyperkinetic movement disorders.

**Highlights:**

To review the most relevant lexicon related to genetic diagnosis, approach to gene testing, testing selection, and complex genetic findings in genetic hyperkinetic movement disorders.

## Introduction

Genetic analyses have significantly evolved over the past few decades, expanding diagnostic opportunities but also increasing the complexity of result interpretation. In the past, genetic testing relied on the sequential sequencing of single genes associated with specific phenotypes. Today, multigene panels, whole-exome sequencing (WES), and whole-genome sequencing (WGS) have become widely available and, in most settings, cost-effective first-line options. These methods offer broader coverage but pose new challenges for test selection and interpretation, particularly in the context of movement disorders. In this review, we aim to clarify key aspects of genetic testing in hyperkinetic movement disorders. We focus on the terminology commonly used to describe genetic variants, the main differences among current sequencing technologies, and practical guidance for navigating the diagnostic process – from selecting the appropriate test to interpreting the results.

## Genetic Variants in hyperkinetic movement disorders

Recent genetic discoveries have revealed that various types of genetic variants are associated with hyperkinetic movement disorders, as summarized in Table 1 [1,2,3,4].

**Table 1:** **Nomenclature of genetic variants**. A definition and examples are reported for the main genetic variants described in the manuscript. A: adenine; C: cytosine; G: guanine; T: thymidine. In the absence of a universal consensus on nomenclature, we adopt the following operational definitions for this review: small insertions, deletions, or combined changes involving fewer than 50 base pairs (bp) are referred to as “del,” “ins,” or “delins”; changes involving more than 50 bp are referred to as copy number variants (CNVs).


GENETIC VARIANT	DEFINITION	EXAMPLE

***Single nucleotide variant (SNV)***	A variant involving one nucleotide, which can be replaced by one other nucleotide, resulting in coding for a different amino acid (*missense variant*) or in a stop codon (*nonsense variant*)	c.2345A>C (nucleotide in position 2345 (A) is substituted with a C)

***Deletions and/or insertions (del, ins, delins or indels)***	Insertions and/or deletions of ≥1 consecutive nucleotides in genomic DNA	Ins: c.2345_2346insG (G inserted between position 2345 and 2346); Del: c.2345_2346delAC (nucleotides between position 2345 and 2346 (AC) are deleted); Delins: c.2345_2346delinsTG (Nucleotides from position c.2345 to c.2346 (AC) are deleted and replaced by TG)

***Duplications***	A copy of ≥1 nucleotides inserted 3’ of the original sequence	Dupl: c.2345_2349dupl (nucleotides between 2345 and 2349 are duplicated)

***Repeat expansions***	A sequence where ≥1 nucleotides is present several times, one after the other, in a region of the DNA prone to expand (e.g., repeats of 3 nucleotides “triplets”, of 4 “quadruplets”, of 5 “pentanucleotides” and so on)	c.2345–2405CAG[45]GGA[1] (between nucleotide 2345 and 2405 there are 45 CAG repeats and 1 GAA repeats)

***Copy number variants (CNV)***	Large stretch of DNA that can be deleted, inserted or amplified – not defined precisely by size, but usually covering at least an exon of a gene or 1,000 nucleotides or more	*PRKN*: exon 2 del (exon 2 of the *PRKN* gene is deleted)

***Structural variants (SVs)***	Complex rearrangement of the DNA through inversion, translocation, gene fusions or CNV	Gene inversion: c.108_205inv (nucleotide between position 108 and 205 are inverted)


**Figure 1:**
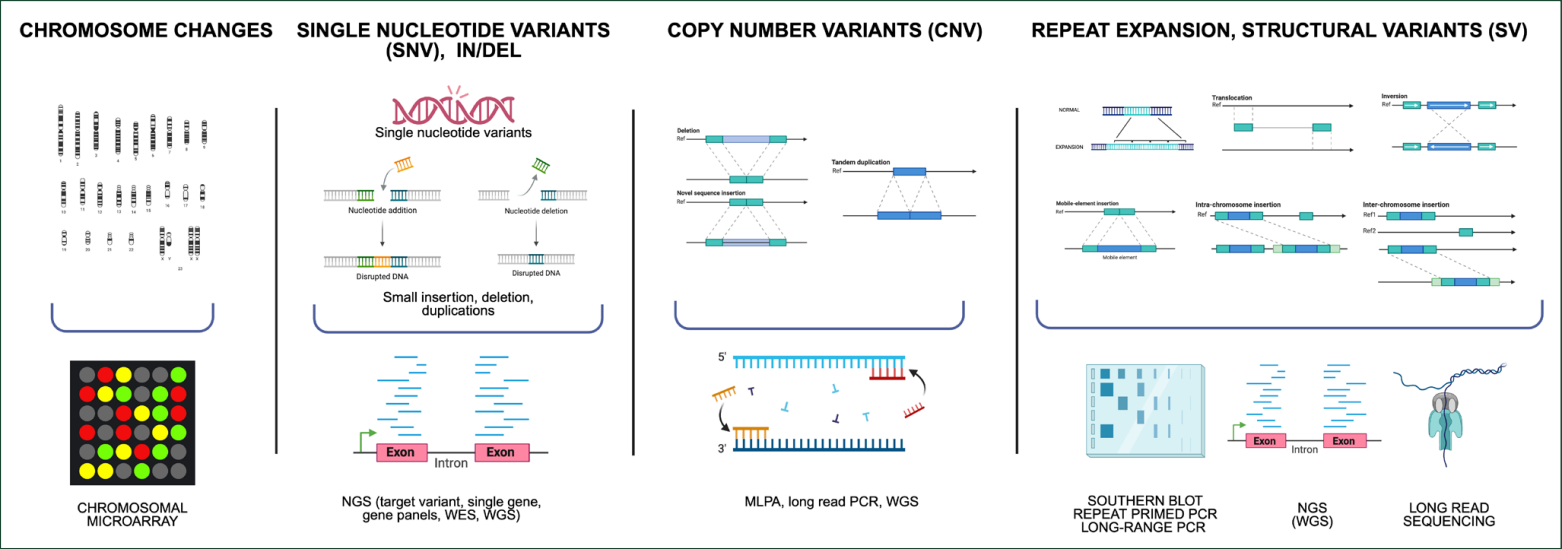
**Schematic representation of genetic changes and appropriate genetic tests**. The four sections of the figure depict the most important genetic variants that can be found in hyperkinetic movement disorders (upper part of the figure) and the appropriate tests to assess each genetic change (lower part of the figure). MLPA: Multiplex ligation-dependent probe amplification; NGS: next generation sequencing; WES: whole exome sequencing; WGS: whole genome sequencing. Figure created with BioRender.

Different types of mutations require specific genetic methods for detection (Figure 1). Therefore, understanding the range of gene variants involved in each clinical presentation – as well as the capabilities and limitations of available testing methods – is essential for accurate diagnosis and for minimizing the risk of missed findings. In the absence of a universal consensus on nomenclature, we adopt the following operational definitions for this review: small insertions, deletions, or combined changes involving fewer than 50 base pairs (bp) are referred to as “del,” “ins,” or “delins”; changes involving more than 50 bp are referred to as copy number variants (CNVs).

## Transition from First to Second and Third Generation Sequencing

The discovery of new genes and the increasing complexity of genotype–phenotype correlations have limited the usefulness of single-gene testing in hyperkinetic movement disorders. A single gene can be associated with multiple clinical presentations (phenotypic pleiotropy – e.g. *ADCY5* gene variants that can manifest with chorea, dystonia and myoclonus in variable association, or *ANO3* and *GNAL* gene variants, previously classified as isolated dystonia, can also present with dystonia and myoclonus), while similar phenotypes may result from variants in different genes (genetic heterogeneity – e.g. cerebellar ataxia, myoclonus epilepsy syndrome etc.).

Sanger sequencing, developed in 1977, remains the gold standard for accuracy and reliability but is inefficient for analyzing multiple genes simultaneously. Its role is now largely restricted to confirming variants identified by more advanced methods.

To address these limitations, Next Generation Sequencing (NGS) was introduced in 2005 [5]. Unlike Sanger, NGS enables high-throughput sequencing of short DNA fragments of DNA (50–300 bases) that can then be bioinformatically aligned to a reference genome for sequence changes (variants) detection [6]. NGS can be used to run gene panels as well as for the analysis of whole exome or whole genome sequencing (WES and WGS, respectively). Multigene panels target a selected group of genes related to a specific phenotype (e.g., dystonia). These panels are curated and validated by laboratories, offering high coverage and often including noncoding or regulatory regions sequencing (such as promoter regions) as well as other additional methods of analyses (such as integration with deletion/duplication analysis for some genes) [7]. WES sequences all the protein coding regions of DNA (exons), around 20,000 genes, representing 1–2% of the genome, while WGS captures both coding and noncoding (intronic) regions. These approaches are especially useful in complex or atypical clinical presentations and in novel gene discovery. As we now know that many pathogenic changes can be found in intronic regions, WGS has become a very important test in the scenario of genetic movement disorders. Nevertheless, intronic variants in the first few nucleotides before or after each exon (usually representing splicing regulatory regions) can also be detected by WES.

NGS-based methods (multigene panels, WES, WGS) can identify missense variants, as well as *del, ins* and *delins*. However, they are often unable to detect certain genetic variants, such as CNV, structural variants (SVs) and repeat expansions.

CNVs are typically identified through multiplex ligation-dependent probe amplification (MLPA). Some larger CNV (from 1 kilobase to multiple megabases to potentially an entire chromosome) and some SVs are detected with chromosomal microarrays analysis (CMA), a molecular genetic test available since 2004, which is commonly advised as the initial diagnostic assessment for individuals presenting with developmental delay, intellectual disability, multiple congenital anomalies, and autism (Figure 1) [7]. Repeat expansions instead require specific methods like Southern blot techniques [8,9,10], repeat-primed PCR (RP-PCR), or long-range PCR (LR-PCR) amplicons. RP-PCR enables efficient amplification of triplet repeats, allowing rapid detection of large pathogenic expansions that are not amplifiable with standard flanking primers. However, it does not provide repeat sizing. Approximate repeat size can be obtained through expanded repeat analysis methods such as LR PCR sized by gel electrophoresis and Southern blotting. The upper detection limit of repeat size detected may vary depending on assay design, laboratory protocols, sample quality, or allele competition during amplification.

Compared to gene panels and WES, nowadays WGS can detect many of these variants (single nucleotide variant (SNV), deletions and/or insertions, copy number variant (CNV), repeat expansions) with proper bioinformatics pipelines. Moreover, new technologies that sequence longer DNA fragments allow for the detection of genetic expansions (both exonic and intronic) and complex rearrangements that may be missed by traditional sequencing methods. This includes third-generation sequencing technologies, which enable single-molecule real-time sequencing of long reads (long-read sequencing, LRS), such as those offered by Oxford Nanopore and PacBio platforms [11]. Currently, these approaches are available only in research settings.

## The diagnostic process from phenotype to genetic test

Given the complexity of gene variants and the growing availability of genetic testing, it is essential for movement disorder specialists to integrate clinical expertise with a working understanding of genetic principles. Genetic tests are diagnostic tools, not standalone answers, and their interpretation should always be anchored to clinical phenomenology.

A careful phenotypic assessment remains the cornerstone of the diagnostic process. This includes: 1) identifying the predominant movement (e.g. dystonia, chorea, myoclonus etc.); 2) recognizing additional neurological or systemic features that may point toward a syndromic presentation; 3) determining the age of onset, which often narrows the list of likely genetic causes; 4) evaluating family history, which may provide clues about the mode of inheritance – often, though not always, characteristic of a given disorder (autosomal dominant, autosomal recessive, X-linked, or mitochondrial).

In parallel, some basic genetic literacy is essential to choose the appropriate test and correctly interpret its results. Specific genetic clues such as population-specific prevalence of some variants, reduced or incomplete penetrance, intrafamilial variability, somatic mosaicism, or skewed X-inactivation, can all influence the diagnostic process. In rare cases, digenic inheritance may also be involved.

Once the appropriate test has been chosen, clinicians should ensure that patients receive clear and comprehensive counseling. The potential benefits of genetic testing – clarifying the underlying cause of the disorder, informing prognosis, and predicting treatment response – should be weighed alongside potential repercussions on family members and insurance coverage. Informed consent is a critical step before proceeding with testing.

Herein we described the main genetic causes of hyperkinetic movement disorders, focusing on the types of genetic variants involved and the most appropriate testing strategies to consider. We cover chorea, dystonia, myoclonus and ataxia. Ataxia is included due to its frequent overlap with other hyperkinetic phenotypes. In contrast, tremor and tics given their limited genetic associations to date are excluded, as findings remain largely within the realm of research. We focus the main discussion on complex or representative genetic cases, and include a broader list of monogenic causes, along with relevant clinical and genetic features in Supplementary Table 1–5.

## Methods

Our approach combines a focused review of the literature with the expert opinion of the authors. In this section, we describe the criteria used to select the genes included in the thematic tables and discussed in the main text. Given the growing number of monogenic movement disorders reported in recent years, our goal was to prioritize clinically relevant genes to support diagnostic decision-making. Selection strategies were tailored to each movement disorder category, aiming to balance completeness with clinical utility.

For hereditary chorea, selection was based on the updated list of genetic causes compiled by the International Parkinson and Movement Disorder Society (unpublished data, MDS Supplementary Table 12, 2023). We prioritized genes in which chorea is the dominant or defining motor feature, and included additional genes when chorea is a core or early element of the phenotype. The full list is presented in Supplementary Table 1.

For dystonia, we followed the latest classification proposed in 2022 by the Study Group for the Nomenclature of Genetic Movement Disorders [29], supplemented by newly reported genes published between 2022 and 2024. These were consolidated into Supplementary Table 2.

For myoclonus, we included the syndromes listed in the consensus paper by the Movement Disorder Society [11], as detailed in Supplementary Table 4. For the purposes of this review, we selected only those syndromes in which myoclonus is the predominant feature. In contrast, epileptic syndromes associated with myoclonus were considered beyond the scope of this review.

We also reviewed the literature on the various forms of Familial Cortical Tremor, given the relevance of this disease group within the field of movement disorders.

Regarding ataxia, we included the forms listed in the consensus paper on autosomal recessive ataxias [12], along with all reported autosomal dominant spinocerebellar ataxias (SCA). While Supplementary Table 3 provides a comprehensive overview of all genetic ataxias, in the main text we focused on those forms that pose the greatest diagnostic challenges.

**Figure 2:**
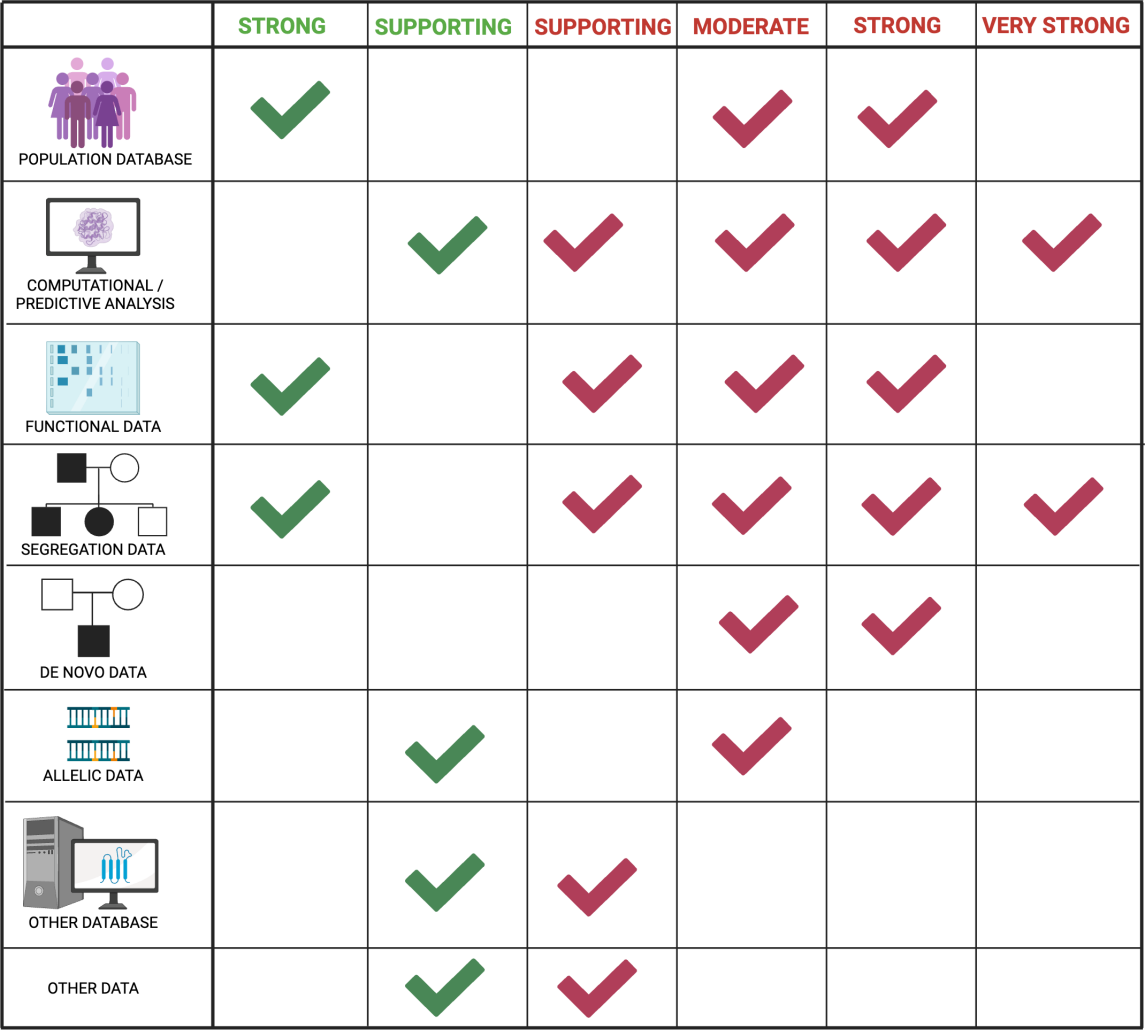
**ACMG variant classification framework: categories of evidence contributing to pathogenic vs. benign interpretation**. Each type of evidence shown in the figure may support either pathogenicity (green) or benignity (red), depending on the data available. **Population data** support pathogenicity when the variant is absent or extremely rare in databases such as gnomAD or ExAC (e.g., PM2), and benignity when the variant is too frequent to be compatible with disease (e.g., BA1, BS1). **Segregation** analysis supports pathogenicity when the variant co-segregates with disease within affected family members (PP1), and benignity when found in unaffected relatives (BS4). **In silico prediction tools** such as SIFT, PolyPhen-2, and CADD support pathogenicity when multiple tools predict a damaging effect (PP3), and benignity when predictions are consistently benign (BP4). **Functional studies** provide support for pathogenicity when validated assays show loss of function or abnormal splicing (PS3), and support for benignity when normal function is demonstrated (BS3). **Allelic data** support pathogenicity when other known pathogenic variants affect the same residue or domain (PM5), and benignity when the variant occurs in *-trans* (on the other allele) with a pathogenic variant but the individual is unaffected (BP2). **Phenotype consistency** supports pathogenicity when the patient’s phenotype strongly matches the gene-disease relationship (PP4), and benignity when inconsistent or unrelated (BP5). **De novo data** contributes to pathogenicity when confirmed by parental testing in a consistent phenotype (PS2), while isolated or unconfirmed *de novo* findings in unrelated phenotypes may provide weak or no support. **Other database or case-level data** support pathogenicity when the variant is consistently reported as pathogenic in databases like ClinVar or HGMD (PP5), and benignity when consistently reported as benign without conflicting evidence (BP6). *Adapted from Richards et al., Genet Med 2015, and updates by ClinGen SVI Working Group, Genet Med 2020*.

Figures were drawn with bioRender (Figures 1 and 2).

## Tips and tricks for the diagnosis of genetic hyperkinetic movement disorders

### Chorea

In genetic chorea, two key diagnostic clues are age at onset and inheritance pattern. Huntington’s disease (HD) is the most common cause of adult-onset chorea. It was the first disease-associated gene to be mapped to a human chromosome (in 1983), and ten years later, a CAG repeat expansion in exon 1 of the *HTT* gene was identified as the cause of this autosomal dominant disorder [13,14].

Full penetrance is typically seen with ≥40 repeats, while 36–39 repeats fall within a reduced penetrance range [15]. HD typically presents around the age of 40 years and is clinically characterized by the combination of chorea, dementia and psychiatric disturbances. The CAG repeat expansion in the HTT gene affects an unstable DNA region. Repeat length correlates with disease severity and earlier onset, and tends to increase across generations. This is a phenomenon known as *anticipation*, particularly pronounced with paternal transmission due to repeat instability during spermatogenesis. Juvenile-onset HD (before age 20), typically inherited from the father, often presents with parkinsonism rather than chorea. In contrast, late-onset HD (after age 60) is usually milder, with rare cognitive involvement. A downstream sequence variant that converts a CAA to CAG triplet can increase repeat instability and influence penetrance, especially in intermediate alleles (36–39 repeats) [15].

Repeat expansions in the *C9orf72* gene are the second most common genetic cause of chorea in individuals of Caucasian ancestry [16]. These intronic GGGGCC expansions, located in the promoter region between exons 1a and 1b, are classically associated with ALS and FTD, though hyperkinetic features can also occur.

A group of disorders known as “HD phenocopies” – or HD-like (HDL) – closely resemble HD in both phenotype and inheritance. HDL-1, caused by extra octapeptide repeat expansions in *PRNP*, a small gene associated with prion diseases, with an unstable region of 5 tandem octapeptide coding repeats between codons 51 and 91. The pathogenic variants of *PRNP* gene encode for an abnormal prion protein, which is misfolded and also induces misfolding in normal prion proteins, leading to cell death and to a group of called “prion disorders” or “transmissible spongiform encephalopathies”. Prion disorders have been classically associated with three major phenotypes, namely the familial Creutzfeldt-Jakob disease (fCJD), fatal familial insomnia (FFI), and Gerstmann-Sträussler-Scheinker (GSS) syndrome, although there are few other rarer, such as HDL-1. Differently from the chorea phenotype, fCJD, FFI and GSS have also been associated to point mutations in the same gene, leading to an amino-acid substitution or premature stop codon, as well as insertion of additional octapeptide repeats [17]. HDL-2, seen only in individuals of African descent, is caused by heterozygous CAG/CTG repeat expansions (>41 repeats) in the junctophilin-3 (*JPH3*) gene [18]. HDL-3 remains genetically uncharacterized, though mapped to chromosome 4 with presumed autosomal recessive inheritance [19]. HDL-4, or SCA type 17 (SCA17), is due to a CAG/CAA expanded repeats on the TATA box-binding protein (TBP) gene, with full penetrance above > 49 repeats and incomplete penetrance between 41–48. Interestingly, patients with intermediate repeat expansions (41–48 repeats) demonstrate a full penetrance when in combination with heterozygous mutations in the *STUB1* gene (SCA48), consistent with a digenic inheritance [20].

Dentatorubral–pallidoluysian atrophy (DRPLA), described almost exclusively in Japanese populations, with only few reports in African American and Caucasian families [21,22,23], is due to a high number (usually >100) of CAG repeat expansion on the gene ATN1. Repeat size strongly correlates with age at onset and phenotype. It can present as a juvenile form (onset <20 years, >65 repeats) with a progressive myoclonic epilepsy phenotype, or as an adult-onset form (<65 repeats) characterized by ataxia, choreoathetosis, and personality changes, with or without cognitive decline [24].

Late onset chorea may also be seen in the chorea-acanthocytosis and McLeod syndromes. The former is an autosomal recessive disease, caused by SNV or CNV in the *VPS13A* gene (ranging from 260 bp to 37 kb, often involving multiple exons), detectable through NGS combined with targeted deletion/duplication analysis. McLeod syndrome is an X-linked recessive condition due to variants in the *XK* gene, including SNV and, in the 10% of cases, deletions from intragenic to multigene size, typically requiring MLPA to be detected [25,26].

Given the wide range of possible variant types causing adult-onset chorea – including repeat expansions, SNVs, deletions, and CNVs – comprehensive approaches such as multigene panels with integrated methods (e.g., sequencing, MLPA, repeat analysis) or WGS are preferred. WES alone may be insufficient.

Outside of juvenile HD, early-onset genetic choreas presenting in infancy, childhood, or adolescence, are typically linked to exonic SNVs. Exceptionally, in Benign Hereditary Chorea, a form of non-progressive chorea with normal or mildly reduced cognition, both small and large deletions have been also reported downstream of the causative gene *NKX2-1*, which encodes for thyroid transcription factor 1 (TTF-1), a protein with a critical role during organogenesis of the basal ganglia, lungs and thyroid. If sequencing is negative despite strong clinical suspicion, further testing with MLPA, CMA, long-range PCR, or WGS should be considered [27]. Both exonic and intronic (splice site) variants have been reported in this gene, all detectable by WES [28,29].

Intronic splice-affecting variants have also been identified in *ADCY5*-related chorea, an autosomal dominant infantile form with facial involvement, often associated with dystonia and myoclonus, marked fluctuations of symptoms, including paroxysmal attacks especially during awakenings and at night, often improving in adulthood.

SNV in *PDE10A* cause two distinct phenotypes: a dominant (de novo) childhood-onset form with normal cognition and bilateral striatal MRI T2-hyperintense lesions, and a recessive infantile-onset form with developmental delay and normal imaging [30,31]. Pathogenic variants in *GNAO1*-gene are increasingly recognized as a cause of early-onset hyperkinetic movement disorders, including chorea, ballism and dystonia. Although chorea may predominate, the phenotype is often complex and may overlap with developmental delay and epileptic encephalopathy. *GNAO1*-related disorders are caused almost exclusively by heterozygous SNVs, mostly de novo missense mutations. A functional dichotomy has been proposed: gain-of-function variants are typically associated with movement disorders, whereas loss-of-function variants are more often linked to early-onset epileptic encephalopathies. Given this mutational spectrum, targeted SNV analysis via gene panels or whole-exome sequencing is the most appropriate diagnostic approach [32].

### Dystonia

Over the past two decades, major advances in the genetics of dystonia have led to the identification of numerous monogenic forms. Given its clinical and genetic heterogeneity, two key aspects should be considered when evaluating a patient: (1) whether dystonia is isolated, combined with other movement disorders, or part of a complex syndrome with neurological/systemic features; and (2) the age at onset, which ranges from infancy to adulthood. Due to frequent genotype–phenotype overlaps, multigene panels or comprehensive genomic sequencing are generally preferred over single-gene testing. It is important to note, however, that gene content varies across panels.

Most genetic dystonias are caused by SNVs or exon-level deletions/duplications, including intronic variants near splice sites. These are typically detectable via multigene panels or WES. However, CNVs may be missed with standard sequencing and should be assessed with gene-targeted deletion/duplication analyses when clinically indicated. In the following section, we highlight genes of particular relevance based on prevalence or distinctive genetic features.

The most common inherited dystonia is associated with TOR1A variants, accounting for 16–53% of generalized dystonia in the non-Jewish population and up to 90% in the Ashkenazi Jewish due to a founder effect. Inheritance is autosomal dominant with ~35% penetrance. Onset typically occurs in childhood or adolescence, often with mild limb dystonia that begins during writing or walking and may generalize over time. Marked intrafamilial variability is common, with focal dystonia (e.g., writer’s cramp) sometimes being the only manifestation [33]. *TOR1A*-related dystonia generally responds well to bilateral globus pallidus interna deep brain stimulation (GPi-DBS) surgery. The most frequent variant is a 3-bp deletion (c.907_909delGAG) in exon 5. Other exonic SNVs, have been associated with milder, adult-onset focal dystonia. A SNV in exon 4 has been shown to reduce penetrance in carriers of GAG deletion – from 35 to 3% – and may serve as a genetic modifier in testing strategies [34].

Other well-established genes associated with isolated dystonia include *ANO3* and *GNAL*, both typically linked to autosomal dominant, adult-onset craniocervical or segmental dystonia. *ANO3*-related dystonia often presents with prominent tremor and involvement of the laryngeal or facial muscles, while *GNAL* variants more commonly cause cervical dystonia that may spread to adjacent regions. Both conditions show variable expressivity and incomplete penetrance, and may be misdiagnosed if not clinically suspected [35].

*KMT2B* gene is now recognized as one of the most common causes of early-onset generalized dystonia, accounting for ~10% of cases and representing the most frequent form outside Ashkenazi Jew (AJ) ancestry. Inheritance is autosomal dominant, typically (84%) due to de novo mutations. Onset in childhood usually begins in the lower limbs and progresses to generalized dystonia with prominent cervical, cranial, and laryngeal involvement. Mild syndromic features (such as intellectual disability, short stature, dysmorphic traits and psychiatric symptoms) may be present but are not universal, and the condition is still classified as isolated dystonia. DBS has shown substantial benefit in many cases. From a diagnostic standpoint, testing should include sequencing (single gene, panel, or WES) and chromosomal microarray (CMA) to detect larger deletions at 19q13.12, which may not be identified through standard sequencing [36]. If CMA and sequencing are inconclusive, targeted deletion/duplication analysis (e.g., MLPA) is recommended to detect intragenic variants.

GTP cyclohydrolase 1-Deficient Dopa-Responsive Dystonia (DYT-*GCH1*), first described in 1994 [37], is an autosomal dominant combined dystonia, with childhood onset. It typically begins with foot dystonia, followed by parkinsonism with diurnal fluctuations and excellent response to low-dose levodopa, usually without motor complications. Penetrance is higher in females (87%) than in males (35%), possibly due to higher basal levels of GTP cyclohydrolase I activities in males [38]. Phenotypic variability is broad, ranging from severe early-onset presentations resembling cerebral palsy to mild adult-onset focal dystonia or parkinsonism.

DYT-*TAF1* (Lubag syndrome), is an X-linked recessive dystonia-parkinsonism, only described in the Filipino population, particularly on Panay Island, due to a founder haplotype.

It primarily affects men, presenting with parkinsonism followed by generalized dystonia. Female carriers are typically asymptomatic but may develop late-onset parkinsonism, explained by extreme skewing of X-chromosome inactivation (98%:2%) that causes heterozygous mutation carriers to express almost solely the mutated allele, similarly to hemizygously affected man [39]. This disorder arises from five SNVs – designated in the literature as Disease-specific Single-nucleotide Changes (DSC) –1,2,3,10,12; a 48-bp deletion in intron 2; and a SINE-VNTR-Alu (SVA)-type retro-transposition insertion into intron 32 that disrupts normal splicing. SVAs are a recently evolved class of retrotransposons that can alter the regulation of genes both transcriptionally and post-transcriptionally through mechanisms such as binding transcription factors and alternative splicing of transcripts [40,41].

*HPRT1*-related disorders are caused by deficiency of hypoxanthine-guanine phosphoribosyl transferase (HGprt), leading to uric acid overproduction and clinical features such as hyperuricemia, kidney stones, and gout as well as various neurologic and behavioral issues in the most severe phenotypes of Lesch-Nyhan disease (LND), which includes significant motor dysfunction, intellectual disability, and self-injurious behavior. As an X-linked recessive condition, heterozygous females are typically asymptomatic, though rare symptomatic cases have been reported due to skewed (nonrandom) X-chromosome inactivation favoring the mutant allele [42].

Recent discoveries have expanded the genetic landscape of dystonia, identifying novel contributors such as *AOPEP, VPS11, VPS16*, and *EIF2AK2. AOPEP* has been implicated in autosomal recessive generalized dystonia. Patients harbor biallelic loss-of-function variants, including frameshift and nonsense mutations, leading to early-onset progressive dystonia with prominent cranial and cervical involvement [43]. *VPS11* and *VPS16* encode components of the homotypic fusion and vacuole protein sorting (HOPS) complex, essential for lysosomal and autophagosomal fusion. *VPS16* is associated with early-onset generalized dystonia, often accompanied by mild neurodevelopmental features. *VPS11* mutations are associated with broader neurodegenerative phenotypes, including dystonia and myoclonus in some cases, but usually accompanied by spasticity, cerebral atrophy, or peripheral neuropathy [44]. *EIF2AK2*, which encodes the protein kinase PKR involved in the integrated stress response, has been recently associated with early-onset generalized dystonia. Missense variants, both de novo and inherited, can result in constitutive PKR activation, leading to increased phosphorylation of eIF2α and disrupted translational control [45]. These recent findings underscore the genetic heterogeneity of dystonia and the importance of broader sequencing approaches (e.g., WES, WGS), particularly in early-onset or syndromic cases. Since these genes are often missing from standard diagnostic panels, increased awareness is crucial to avoid missed diagnoses.

### Ataxia

Hyperkinetic movement disorders can be frequently observed in primary forms of ataxia. For example, DRPLA may present with ataxia and myoclonus or seizure in pediatric-onset cases, and choreoathetosis in adult-onset cases. Chorea can also be a prominent presentation of SCA17. In progressive myoclonus ataxia, hyperkinesia is typically severe and disabling (see “Myoclonus” section). Tremor is a key feature in FXTAS and SCA48, while episodic forms of ataxia (EA1,2,5,6,9) can be initially misinterpreted as hyperkinetic movement disorders (Supplementary Table 3). Genetic testing for ataxias remains challenging due to ongoing discovery of novel forms. A detailed family history and inheritance pattern are essential to guide diagnosis. Autosomal dominant ataxias, with adult onset and cerebellar atrophy typically suggest a SCA. Over 40 genes are now linked to SCAS, presenting either as pure cerebellar syndromes or with neuromuscular or cognitive involvement (Supplementary Table 3) [46]. A significant proportion are due to exonic or intronic nucleotide repeat expansions, particularly CAG/polyglutamine repeats, which inversely correlate with age at onset (Supplementary Table 3) [47].

These expansions show paternal anticipation, especially in SCA2, SCA3, and SCA6, with variable geographic prevalence: SCA3 globally, SCA2 in Cuba, Mexico, and Italy, SCA6 in Northern England, SCA1 in Africa, SCA7 in Venezuela, Sweden, and Finland, and SCA10 in Latin America. Founder effects have been proposed based on haplotype analyses [48,49].

Multi-gene panels are commonly the first choice used, but WGS now enables direct detection of repeat expansions, offering a more comprehensive diagnostic tool that also captures SNVs and indels. Similar to HD expansion, also in the case of SCA1 (due to a polyglutamine expansion of the *ATX1* gene), CAT interruptions of the CAG repeats in the intermediate expansion range (between 36 to 46) can determine disease penetrance and the pathogenic role of longer expansion (see Case 3) [50,51]. For this reason, CAT assessment needs to be included in gene testing for SCA1. Guidelines for gene testing in different forms of SCAs have been published in 2016 [52].

SCA6, caused by pathogenic variants in the *CACNA1A* gene, exemplifies genetic pleiotropy. Variants in this gene are also associated with Episodic Ataxia type 2 (EA2) and familial hemiplegic migraine. SCA6 is typically linked to CAG expansions, while frameshift and nonsense variants are more frequent in EA2 (see Supplementary Table 5).

Long-read sequencing technologies have enabled detection of previously undetectable expansions, such as a newly described SCA form due to *THAP11* expansion [53].

Recessive forms of ataxia are usually characterized by either childhood or adult onset, lack of family history, and often associated with additional neurological symptoms. Although the most common form of autosomal recessive ataxia, Friedreich ataxia (FA), is caused by GAA expansion of the Frataxin (*FXN*) gene in most of the cases, the majority of AR ataxias are caused by SNV, small deletions or duplications. While GAA expansions are found in 96% of FA cases, SNV and deletion/duplication have been reported in the rest of the cases. Therefore, appropriate tests should be performed to look for these variants when GAA expansions are found in only one allele in subjects with phenotype very consistent with FA. It is important to consider that, although being a classical pediatric condition, FA can present also in adulthood in the form of Late Onset FA (LOFA) with onset between the age of 25 and 40 and Very Late Onset FA (VLOFA) with onset after the age of 40 years. These patients usually present a shorter GAA expansion compared to FA cases in at least one of the two alleles [54]. NGS-based tests (gene panels, WES or WGS) are essential in identifying causative variants.

Late onset forms of progressive ataxia are usually more difficult to genetically diagnose. However, in the last few years, intronic expansions in the *RFC1* and *FGF14* genes have been found in a number of the patients with late onset cerebellar ataxia (LOCA), respectively in the cerebellar ataxia neuropathy and vestibular areflexia syndrome (CANVAS) and SCA27B [55,56,57]. The identification of genetic variants associated with these phenotypes has been favored by the recent growing development of the sequencing technologies including WGS and LRS which have allowed the identification of these intronic expansions and are now critical tools in resolving previously unsolved ataxias.

### Myoclonus

Genetics of myoclonus is complex and still not fully defined [11]. Myoclonus is often part of more complex conditions and frequently associated with epilepsy. For this reason, genetic panels for isolated myoclonic syndromes are rarely available in diagnostic laboratories.

The majority of the myoclonic syndromes present in childhood. When seizures are prominent, a comprehensive gene panel for epileptic syndromes or WES should be considered. Of note, when dysmorphic features and developmental delay are found, microarray analysis should also be performed.

An important group of myoclonic syndromes is represented by the progressive myoclonus-ataxia (PMA) and progressive myoclonus-epilepsy (PME or EPM) (Supplementary Table 4). These are both characterized by a childhood onset, progressive syndrome, with different degrees of myoclonus, ataxia, epilepsy, as well as cognitive impairment, especially in the context of PME. These conditions are important to suspect since many of them are caused by genetic variants that would be missed by NGS analysis. Indeed, conditions such Unverricht-Lundborg disease (EPM1) and DRPLA are caused by exonic expansion in the corresponding genes, to be tested through expansion panels or WGS (Supplementary Table 4). In EPM1 patients, the majority of genetic variants is caused by homozygous expansion of a dodecamer (CCC-CGC-CCC-GCG) in the *CSTB* gene (90% of cases), while 10% of patients present compound heterozygous variants with a pathogenic dodecamer expansion on one allele and SNV or insertion or deletion (i.e. indels). Interestingly, compound heterozygous variants have been associated with a more severe phenotype and may be more frequent in the male population [58,59,60]. Thus, all these types of variants should be assessed with appropriate tests when EPM1 is suspected. More recently, homozygous frameshift or stop codon variants have been found in patients with a severe phenotype characterized by infantile severe onset with developmental delay, demyelination, and microcephaly with onset in infancy [61,62].

On the other side, the Myoclonic epilepsy with ragged-red fibers (MERRF) is caused by mitochondrial DNA variants, thus sequencing of the mtDNA should be included in the diagnostic workup. Maternal inheritance, due to inheritance of mtDNA only through the maternal germline, and heteroplasmy (the coexistence of different mtDNA variants within a single cell, whose levels can vary considerably between cells, organs or even individuals) pose major challenges for the diagnosis of mtDNA disease.

Sequencing of the mtDNA can be requested as part of WES analysis, or as a separate test. In 80% of cases, this condition is caused by the m.8344A>G variant. Like the other mitochondrial disorders, heteroplasmy should be considered. Heteroplasmy indicates the fact the different tissues can carry different ratios between mitochondria with and without a pathogenic. For this reason, when a test on peripheral blood (i.e., peripheral leukocytes) is negative, testing other tissues (such as urine, muscle, fibroblast from the skin) should be considered.

Myoclonus can also be associated with dystonia in the myoclonus-dystonia syndrome caused by variants in the *SGCE* gene (*SGCE*-MD), classically proximal, subcortical myoclonus with a characteristic “shivering” component, appendicular myoclonus, possible spasmodic dysphonia, a strong positive response to alcohol, and a history of anxiety and obsessive-compulsive disorders (OCD) (Supplementary Table 4). Importantly, this condition is inherited in a patrilinear fashion due to maternal methylation in the presence of a SNV that silences its expression [63]. This mechanism is escaped in 5% of cases, where families may present a matrilinear transmission as well. *SGCE*-MD is more rarely associated with microdeletion 7q21, with a variety of additional features including short stature, facial dysmorphism, intrauterine growth deficiency, microcephaly, cognitive impairment, language delay, psychosis, joint laxity, and bone fractures [64]. *SGCE-MD* phenotype is also observed in the Russell-Silver syndrome where abnormal methylation of the Chr. 11p15.5 (which includes the *SGCE* gene) or maternal uniparental disomy of chromosome 7 (upd(7)mat) can be present. In these patients, the myoclonus-dystonia is associated with a characteristic growth restriction and dysmorphic features. Karyotype, methylation analysis and microarray analysis can be required in these cases [65]. A combination of dystonia and myoclonus can also be associated with other genes, including but limited to *KCDT17, KCNN2, ATP1A3, YY1, ANO3, ADCY5*, although these forms often lack the hallmark features of the SGCE-MD syndrome (such as a proximal and shivering myoclonus, paternal imprinting, and exquisite response to alcohol) [66].

Intragenic deletions and duplications can be found in the *EPM2A* and *NHLRC1* (EPM2) genes, *ASAH1* gene (SMA-PME), *NUS1*, and *SGCE* (myoclonus-dystonia). In order to detect these variants, MLPA, LRS, long-range PCR, as well as gene target microarray should be considered.

Special consideration should also be given for Gaucher disease type 3 (GD3). Missense, nonsense, small intragenic deletions and/or insertions, and splice site variants reported in the *GBA1* gene can be assessed with the NGS technologies. However, due to the presence of a pseudogene with a high degree of homology (GBAP1, > 90% homology), on one side complex gene-pseudogene rearrangements are also reported, on the other side erroneous sequencing of the pseudogene can occur. For this reason, PCR-based methods using primers targeting only the *GBA1* gene have been developed by most of the labs. LRS or bioinformatic tools for the analysis of WGS data represent a valid alternative [67,68,69].

The Progressive Ataxia with Palatal tremor syndrome (PAPT) is still a difficult entity to diagnose genetically. Pathogenic variants in the *GFAP, NF, POLG, GM2, CTX* genes have been reported in a few patients [70,71,72,73,74]. A specific type of PAPT is the one associated with SCA20, described in a large Anglo-Celtic family from Australia, where palatal myoclonus and ataxia were associated with spasmodic dysphonia, dysarthria, and brain calcifications [72]. Genetic analysis pointed to a duplication in the chromosome 11, although the causal gene has not been isolated yet among the ones included in this region [75].

Finally, adult-onset myoclonus, with or without epilepsy in the family, can be suggestive of a form of familial cortical tremor or cortical myoclonus (FAME). While different specific genes were prioritized across different families with this phenotype, more recent studies leveraging PCR-based or LRS were able to detect pathogenic intronic expansions in the targeted genes [76]. These expansions mostly consist of different arrangements of TTTTA and TTTCA motifs, although the understanding of the pathogenic mechanisms related to these expansions and their structure is still ongoing [76]. Consequently, most of the genetic analysis for FAME-associated genes are still research based and not available in diagnostic laboratories.

### Challenges and implication of result interpretation

Genetic tests whether single gene testing, multi-gene panels, mtDNA sequencing, WES/WGS, or long read sequencing can identify a wide range of variants, including SNV, deletions and/or insertions, duplications, repeat expansions, CNV and complex rearrangements. These variants are interpreted according to the American College of Genetics and Genomics (ACMG) [77]. The ACMG classification system integrates multiple lines of evidence, including: 1) population frequency data, from large control databases (e.g., *gnomAD, ExAC, 1000 Genomes Project*); 2) computational (in silico) predictions of pathogenicity (e.g., *SIFT, PolyPhen-2, CADD, REVEL –* summarized in [78]); 3) segregation studies (i.e., variant presence in affected family members and absent in unaffected ones); 4) functional studies, such as enzymatic activity assays, minigene splicing assays, subcellular localization studies (e.g., immunofluorescence), or rescue experiments in model systems (e.g., small animal models, cell lines, or patient-derived cell models), to be performed according to established protocols and with disease-relevant models, such assays are considered a reliable source of evidence; 5) consistency of the allelic phase with the mode of inheritance (monoallelic variants for autosomal dominant conditions and biallelic for recessive conditions) or presence of *de novo* variants (when WES/WGS is performed on the proband only, phase can be accessed via family testing and segregation analyses, or leveraging more advanced sequencing techniques such as LRS); 6) information from other relevant databases; 7) phenotype concordance (Figure 2). Within these categories, pathogenic (P) or benign (B) very strong (VS), strong (S), moderate (M), or supportive (P) criteria are identified. These data are weighted in a semi-quantitative scoring system to classify variants as benign, likely benign, variant of uncertain significance (VUS), likely pathogenic, or pathogenic. Variants that are classified as benign or likely benign are usually not reported in the context of clinical genetic tests.

To better assess VUS, other relevant strategies can be employed to understand their potential clinical relevance. First, evaluate whether the variant is consistent with the patient’s phenotype. This includes not only clinical symptoms, but also characteristic brain MRI (e.g., metal deposition in Neurodegeneration with Brain Iron Accumulation (NBIA), Primary Familial Brain Calcification (PFBC), hypermagnesemia, or Wilson disease; hyperintensities in the middle cerebellar peduncle in FXTAS, or caudate atrophy in HD), laboratory abnormalities (i.e. increased alpha-fetoprotein in ataxia telangiectasia (AT), absent ceruloplasmin in Aceruloplasminemia, decreased ceruloplasmin and copper in Wilson disease, absent expression of the Kx erythrocyte antigen in McLeod syndrome, or distinctive cerebrospinal fluid (CSF) neurotransmitter profiles), and disease-specific epigenetic signatures (i.e. in *KMT2B*-dystonia) [79]. Second, gene expression in disease-relevant tissue or correlation of the identified gene with known disease molecular pathways can be suggestive of pathogenicity. Third, assessing tolerance of by assessing the protein tolerance to certain type of variants according to dedicated scores (Z-score ≥ 3 for missense variants and pLI socre ≥0. 9 for loss of function variants) can help in variant prioritization. Finally, reassess the variant over time by consulting publicly available databases, such as ClinVar database (https://www.ncbi.nlm.nih.gov/clinvar/), which is continuously updated with new submissions from research groups and clinical laboratories. Additional information from the literature describing similar variants in patients with overlapping phenotypes may also support reclassification of a VUS.

Effective use of these tools requires close collaboration between clinicians, geneticists, genetic counselors, and testing laboratories. Such collaboration is key to interpreting findings and determining appropriate next steps, including additional testing, family studies, or reanalysis of sequencing data.

Another crucial element in variant interpretation is prioritization based on phenotype. In clinical WES or WGS, only variants classified as pathogenic, likely pathogenic, or VUS in phenotype-relevant genes are reported. Therefore, providing a thorough and accurate description of the patient’s phenotype at the time of testing is essential. Follow-up with genetic laboratories – typically every 1–2 years – may enable reinterpretation of previously reported VUS or detection of newly discovered pathogenic variants. As knowledge evolves, previously negative tests may eventually yield a diagnosis.

In some cases, additional genetic findings can help to clarify the pathogenicity of a variant. For instance, *STUB1* variants (SCA48) may modify the penetrance of intermediate-length expansion in the *TBP* gene associated with SCA17 (see “Chorea” section). Another example is the presence of sequence interruptions (“brakes”) in polyglutamine expansions, which may reduce pathogenicity in alleles falling within the so-called gray zones [80] (see “Ataxia” section). These findings are usually included in the reports from genetic laboratories.

Finally, it is crucial to inform patients that genetic results can have significant implications for other family members. For example, autosomal dominant conditions with full penetrance (such as HD) can affect the risk profile of the proband’s siblings or offspring. For this reason, genetic testing should always be performed in appropriate settings, that provide pre- and post-test counseling, appropriate psychological assessments (if needed) and access to psychological support and expert consultation [81].

### Common pitfalls in genetic testing and interpretation

Despite the increasing availability and sophistication of genetic testing technologies, several recurrent pitfalls can limit their diagnostic yield and interpretation. One major limitation is the lack of detailed clinical information, which can prevent the accurate correlation between phenotype and genotype, particularly in the context of agnostic approaches such as WGS. Comprehensive phenotyping (including neurological, systemic, imaging and biochemical data) is essential to guide variant prioritization and interpretation.

Another common issue is inappropriate test selection. When using targeted panels, it is crucial to match the test content with the patient’s clinical presentation (“phenomenology first”). Selecting overly broad or overly narrow panels without careful clinical correlation may lead to missed diagnoses or identification of non-informative variants.

Even whole genome sequencing (WGS) has its limitations. Certain pathogenic variants can be missed due to current technological or bioinformatic constraints. These include copy number variants (CNV), deep intronic changes, non-canonical splice site variants, and promoter or regulatory region mutations that are not consistently detected or prioritized by standard pipelines.

Lastly, tissue mosaicism represents an under-recognized cause of false-negative results, especially in disorders caused by postzygotic mutations. When mosaicism is suspected based on phenotype or inheritance pattern, testing of multiple tissues or highly sensitive methods may be warranted.

Raising awareness of these pitfalls and integrating clinical expertise at every step from test selection to variant interpretation is critical to maximize diagnostic accuracy and patient care.

### The role of research in the diagnosis of genetic hyperkinetic movement disorders

Although major advances have been made in the field of genetic movement disorders over the past decades, research continues to play a crucial role in identifying new genes and novel variant mechanisms. Recent reviews suggest that current sequencing and testing methods can detect a causative genetic variant in certain cases in up to 60% patient with movement disorders, typically through multi-step approaches (including but not limited to single gene, gene panels, MLPA, WES, WGS) [82,83,84]. This means that a significant number of patients remain undiagnosed despite extensive genetic tests.

There are now numerous research programs and laboratories worldwide dedicated to undiagnosed genetic disease worldwide, some specifically focused on movement disorders. These programs leverage advanced technologies and collaborative networks to identify new genes or new or cryptic variants in known genes that may be missed by standard clinical methods. Thanks to tools such as GeneMatcher, which facilitates the sharing of newly identified candidate genetic variants among researchers to enable phenotype matching and assess pathogenicity, and thanks to the strengthening of international collaborations, these programs are gradually reaching resource-limited settings. However, broader access is still needed [85].

On the other side, with the development of easily approachable sequencing technologies (i.e., transcriptomics, epigenomics, metabolomics and proteomics) and the growing availability of disease-relevant tissue modeling though induced pluripotent stem cell technologies, multi-omics approaches have become increasingly relevant in the context of gene discovery. Specifically in the field of movement disorders, combining DNA sequencing with RNA sequencing (either on fibroblast or on target tissues), proteomics and metabolomics has contributed to the identification of new disease causative genes or variants supporting in gene or variant prioritization [86,87,88,89].

Finally, new and advanced sequencing methods capable of scanning previously inaccessible or complex genomic regions at higher resolution will likely allow the identification of new genetic diagnoses. This is the case of the identification of the intronic expansion that causes FAME or SCA27B [56,90,91] (see session on “Myoclonus” and “Ataxia”). In particular, long read sequencing technology, has allowed uncovering previously missed complex structural variants or intronic expansion, as well as allowing to study DNA sequencing and methylation profiles in the same runs [92].

Diagnostic testing for some of these conditions is still not available and assessments are still only research based. On the contrary, other research-based genetic discoveries, are now becoming part of the routine diagnostic process (i.e., SCA27B) [93].

This evolving scenario highlights the continuum between research and clinical diagnostics, where expert clinician-geneticists play an important role in advancing the field and translating discoveries into patient care.

## How to approach a clinical case?

### Choosing the right test

***Case history*:** A 65-year-old Caucasian man presented with a progressive worsening of balance and frequent falls. About 15 years prior he started developing spells of dizziness, blurred vision, and poor balance, lasting a few minutes. In between episodes he was feeling normal and his neurological exam was unremarkable. In the last three years symptoms became constant and started to progress. He had no family history of similar symptoms or of other neurological disorders. He had not been taking any chronic medications except for simvastatin for hypercholesterolemia.

His exam showed a scanned speech, ocular nystagmus, mostly downbeat, appendicular dysmetria and an ataxic gait with a wide base.

***Assessments*:** Clinical history is suggestive of a progressive cerebellar condition. The absence of exposure to medications (such as phenytoin), solvent, as well as the prolonged clinical history (more than 15 years) would rule out secondary causes or infectious and paraneoplastic conditions, while suggesting a possible genetic cause. Neurodegenerative conditions, such as Multiple System Atrophy (MSA) should be considered as well. A brain MRI showed a moderate cerebellar atrophy, disproportionate for age. Blood work was unrevealing, including vitamin E and vitamin B12. Dysautonomia was not present, as well as no MRI suggestive features (i.e., the “hot cross bun” sign), pointing against the diagnosis of MSA. Considering the negative family history, a recessive condition or a *de novo* variant should be suspected. To assess genetic causes in this patient, an ataxia panel could be considered. However, while the majority of autosomal recessive forms of Ataxia (except for FA) would be captured but NGS analysis, autosomal dominant SCA due to exonic and intronic repeat expansion, would require a different approach (i.e., an expansion panel, long read PCR, Southern blot). Considering subject age (adult onset) and the combination of episodic onset and downbeat nystagmus, the diagnosis of SCA27B, an autosomal dominant condition due to an intronic repeat expansion, is high in the differential. WGS is now able to test all the AR forms of ataxia (mostly SNV) as well as exonic and intronic expansions. Considering the broad differential for this patient and the possibility of SNV or repeat expansion analysis, WGS was performed.

***Results interpretation*:** Proband only WGS revealed a heterozygous GAA intronic expansion (>300 expansion) in the intron 1 of the *FGF14* gene consistent with the diagnosis of SCA27B. This region of the DNA in the *FGF14* gene is prone to expansion and instability. The combination of a reduced penetration and the instability of the expansion between generations, can manifest with an absence of family history for subjects affected with this condition. Expansions in the range of 250–300 have a reduced penetrance, and a diagnosis of SCA27B can be established if patients present consistent symptoms and/or segregation in the family. As we learn more and more about this condition, the establishment of this diagnosis can be informative about progression of the symptoms. Also, it has been reported that subjects with SCA27B can benefit from the treatment with 4-diaminopyridine [93]. Finally, after the establishment of this diagnosis, genetic counseling for family members can be offered. In particular, offspring of this subject have a 50% of inheriting the expanded allele. It has been reported that expansion has a tendency to expand with transmission to females and to contract during transmission to males [56]. Therefore, offspring may present expansion in the reduced penetrance range and do not manifest symptoms.

### Interpreting a VUS

***Case history*:** A 28-year-old African-American woman presents to the clinic for a history of generalized epilepsy, mild developmental delay and occasional falls. Her exam showed myoclonus in the face and upper body, mostly affecting her hands with actions but also at rest, as well as mild ataxia in the upper limbs and of her gait. Her history was characterized by the onset of “shakiness” (most likely myoclonus) during her first decade, followed by generalized tonic-clonic seizure and progressive difficulties attending school. There was no family history of seizure or any other neurological disorders. There was no history of consanguinity in the family. Brain MRI was unremarkable except for mild cerebellar atrophy. There was no history of other clinical conditions.

***Assessments*:** The history of an early onset epilepsy associated with ataxia and myoclonus not related to perinatal insults, is highly suggestive for a genetic condition. Epilepsy represents the main symptom in this patient, and the combination with myoclonus, ataxia and mild intellectual delay are consistent with a form of PME or PMA. There are a large number of conditions associated with epileptic syndromes, in most cases consisting of SNV or *insertions and/or deletions*, that can be tested through next generation sequencing including either gene panels, as well as WES or WGS. However, some forms of PME and PMA are caused by exonic expansions (such as in the case of EPM1 and DRPLA). In order to cover for these two conditions, separate panels assessing gene expansion (either through Southern blot, RP-PCR, LR-PCR or LRS technologies) should be performed. WGS offers the possibility to cover for all the mentioned conditions and genetic changes, and it would be the most comprehensive test in this case. In this case, WES to test for epileptic syndromes, was initially performed.

***Results interpretation*:** Genetic analysis in the proband revealed a heterozygous, novel, missense variant in the *NUS1* gene classified as VUS. Heterozygous, pathogenic variants of *NUS1* have been reported in the cases of early onset syndromes characterized by various combinations of myoclonus, ataxia, intellectual disability, with or without epilepsy [94]. Although the phenotype can be consistent with the presentation of this patient, in order to characterize the pathogenic role of the identified variant, additional evidence is required. In particular:

- This variant was absent from large population databases (i.e., gnomAD), supporting for the variant to be rare and thus possible associated with a clinical phenotype (PM2 criteria according to ACMG classification) [77]
- This was a novel missense variant in the same position where a previous reported variant (different amino acid change) was associated with a pathogenic phenotype (PM5 criteria according to ACMG classification) [77]
- Parental testing was performed and it confirmed that the variant was *de novo* (i.e., present only in the proband and not in parents), supporting a segregation of the variant with disease presentation (PM6 criteria –parental testing was not performed in this case)

Because three moderate pieces of evidence of pathogenicity were present (PM2, PM5, PM6), the variant was re-classified as likely pathogenic [77].

### Interpreting complex genetic reports

***Case history*:** A 45-year-old Hispanic man presents to the clinic for gradual worsening slurring of his speech and mild imbalance. He also started noticing that sometimes he has difficulties reaching out to things and coordinating his movements. The symptoms started about 3 years ago and they gradually progressed. He developed difficulties riding a bike and people asked him to repeat himself at times. Blood work was unrevealing and a brain MRI showed a moderate atrophy of the cerebellum. In his family, his father, paternal grandfather, one of his paternal aunts, and his paternal great grandfather had similar symptoms and they all ended up needing a wheelchair by the end of their lives. He had one older sister who was unaffected. His family was from Chile from both the maternal and paternal side and there was no history of consanguinity in the family.

***Assessments*:** A history of an adult-onset progressive cerebellar syndrome (dysarthria, ataxia and dysmetria), with a negative work up and evidenced of cerebellar atrophy on the brain MRI are highly suggestive of a form of Spinocerebellar Ataxia (SCA). The strong family history, consistent with an autosomal dominant inheritance, corroborates this hypothesis. Known SCAs are now more than 40 and they are mostly caused by intronic or exonic expansion. Different forms of SCAs can present with pure cerebellar syndromes or with other associated features that can help distinguish among the different types. In this case, the patient presented with a pure cerebellar syndrome, consistent with SCA1, 2, 3, 6, 17. Although some of these SCAs can present with other neurological features (i.e., slow saccades in SCA2, chorea and psychiatric features in SCA17), they have also been reported in pure cerebellar forms of SCA in some patients. Ethnicity and geography can also help prioritizing testing for these patients. For example, SCA3 is the most frequent form world-wide. Because of possible overlaps between these conditions, an ataxia expansion panel should be considered, making sure that at least all the subtypes listed above are included. Alternatively, nowadays, WGS allows testing for repeat expansion in these genes, with the advance of testing in parallel other forms of AD ataxia due to SNV. In this case, because of the strong family history, his clinical representation, and his ancestry, one of the SCA was strongly suspected and the SCA expansion panel was requested.

***Results interpretation*:** Genetic analysis in the proband reported 29 CAG repeats in one allele in the *ATXN1* gene and 45 repeats in the other allele. The number of repeats in the other genes was within normal limits. Less than 35 CAG repeats in the ATXN1 are within the normal range that is not associated with clinical manifestations, while greater than 47 repeats are considered fully penetrant expansion associated with symptoms of SCA1. Instead, 36 to 46 CAG repeats can variably be associated with clinical manifestations depending on the genetic makeup. In particular, if CAT interruptions are present within the CAG expansion, they will exert a stabilizing effect with no symptom manifestation. When CAT interruptions are not present, 39 to 46 CAG expansions are pathogenic, while 36 to 38 expansions have variable penetrance but can expand in the offspring and become pathogenic.

This patient presented a normal expanded allele (29 CAG repeats) and an intermediate expanded allele (45 CAG repeats). No CAT interruptions were reported. Therefore, this expansion is considered pathogenic and it is consistent with the patient’s clinical manifestations and family history. CAG expansions in the SCA1 gene are more unstable when paternally transmitted and can present increased expansion in the offspring. On the contrary, when the variant is transmitted from the maternal side, the repeat expansion can present contraction and thus reduce the risk of disease.

## Conclusions

Genetics is playing an increasingly central role in the diagnosis of movement disorders, particularly hyperkinetic syndromes. However, selecting the appropriate genetic test and interpreting results can be challenging for clinicians and movement disorder experts. A clearer understanding of the available testing modalities, the spectrum of genetic variants, and their implications is essential to navigate the diagnostic process effectively.

In this rapidly evolving field, ongoing updates and close collaboration with genetics experts will remain crucial. Strengthening these foundations will empower clinicians to approach the genetic evaluation of hyperkinetic movement disorders with greater confidence and precision.

## Additional File

The additional file for this article can be found as follows:

10.5334/tohm.971.s1 Supplementary File. Supplementary Tables 1–5.
