# Supplementary material for: Decoding the Alphabet Soup: A Practical Guide to Genetic Testing in Hyperkinetic Movement Disorders
Source: Tremor Other Hyperkinet Mov (N Y). 2025 Jun 26;15:28. doi: 10.5334/tohm.971 (PMC12203903; doi:10.5334/tohm.971)
Supplement: Supplementary File. — Supplementary Tables 1–5. [file tohm-15-1-971-s1.pdf]

CHOREA

Gene	Condition	Her	Variants	NGS panel	Expansion	WES	WGS	mtDNA	Other	AOO	Genetic tricks	Clinical traits	Ref
HTT	**Huntington disease**	AD	CAG expansion		x		x		LRS	Childhood to late adulthood	Anticipation on paternal inheritance. Penetrance: complete with ≥ 39 rpt; incomplete with 36-38 rpt (somatic mosaicism with possible longer expansions in certain brain areas). Correlation between rpt, AOO and severity. Genetic modifiers like *FAN1, MTMR10, RRM2B, URB5* play a significant role in disease development.	**Classic form**: chorea, dementia, psychiatric symptoms. **Juvenile form** "*Westphal variant*": rigid-hypokinetic, seizures, dementia, psychiatric symptoms. **Brain MRI:** caudate nucleus head atrophy	(1,2)
PRNP	**HDL-1**	AD	Octapeptide expansion		x		x		LRS	Adulthood	Allelic disorder to Creutzfeldt-Jakob disease, fatal familial insomnia, Gerstmann-Straussler disease, Spongiform encephalopathy with neuropsychiatric features, and Cerebral amyloid angiopathy *PRNP*-related (also caused by SNV)	"Early-onset prion disease" with prominent psychiatric features, ataxia, chorea, rigidity, dysarthria, dementia, seizures.	(3)
JPH3	**HDL-2**	AD	CAG/CTG expansion		x		x		LRS	Adulthood	Only described in patients of African ancestry. Anticipation. Penetrance: < 33-35 rpt may be unstable in vertical transmission, 40-43 rpt: incomplete; > 43 rpt complete (impact of alleles of 36-39 rpt remain uncertain)	Psychiatric symptoms, dementia, chorea and/or rigidity/ parkinsonism, dysarthria. **Brain MRI:** cortical and striatal atrophy	(4,5)
genetic cause is unknown	**HDL-3**	AR	*unknown*	NA						Childhood - Adulthood		Normal developmental, followed by decline in cognition and movements, with chorea, dystonia, ataxia, gait instability, spasticity. Seizures. **Brain MRI:** frontal cortical and caudate atrophy	(6)
TBP	**HDL-4, SCA17**	AD (rarer AR)	CAG/CAA repeats		x		x		LRS	Adulthood	Anticipation. Penetrance: complete with >46 rpt; incomplete with 41-46 rpt; unaffected with 25-41. Digenic disease**:** patients with intermediate *TBP* expanded alleles in combination with heterozygous mutations in the *STUB1* gene (SCA48) demonstrate full penetrance	Cerebellar ataxia, chorea and/or rigidity/parkinsonism, pyramidal signs, cognitive impairment, psychosis, seizures **Brain MRI:** cerebellar atrophy	(7–10)
ATN1	**Dentatorubral-pallidoluysian atrophy (DRPLA)**	AD	CAG repeats (100%)		x		x		LRS	Early adulthood	Almost only described in Japanese (few families African Americans and Caucasians). Number rpt correlates with different phenotypes	**Juvenile form** (<20yy, typically >65 rpt): developmental delay, intellectual disability, myoclonus, and epilepsy **Adult-onset form:** ataxia and/or choreoathetosis, personality changes +/- cognitive decline	(11–13)

C9orf72	**Frontotemporal dementia and/or Motor neuron disease**	AD	Hexanucleotide repeats<br>G_4C_2 (GGGGCC)		x		x		LRS	Adulthood	Second most common cause in Caucasians.<br>Repeats size: 25-60 rpt uncertain significance (somatic mosaicism with possible longer expansions in certain brain regions); 61 to >4000 rpt pathogenic.	Movement disorders, including chorea, dystonia, myoclonus, tremor, parkinsonism may precede signs of FTD/ALS, or even be present in isolation	(14)
VPS13A	**Chorea-acanthocytosis**	AR	SNV, del/dup, splice site<br>(70-90%)	x		x	x			Early adulthood	Penetrance is complete.	Triad of progressive movement disorder (chorea, dystonia, tics with prominent orofacial involvement and common bite lesions; in later stages parkinsonism) Cognitive alterations. Psychiatric issues. Epileptic seizures. Myopathy, neuropathy with weak/absent tendon reflexes. Dysphagia, dysarthria. Hepatosplenomegaly. Cardiomyopathy, arrhythmia.<br>**Brain MRI:** caudate atrophy.<br>Muscle and liver enzymes, markers of hemolysis. RBC acanthocytosis	(15)
			CNV				x		del/dup analyses<br>(10-30%)				(16)
XK	**McLeod syndrome**	X-linked recessive	SNV<br>(60%)	x		x	x			Adulthood	Only males affected.<br>Females heterozygous have **mosaicism**: may develop clinical manifestations including chorea or late-onset cognitive decline.<br>Penetrance is almost complete.	**CNS:** Psychiatric issues, cognitive alterations, movement disorders, sensorimotor axonopathy, muscle weakness. **Cardiac**: Dilated cardiomyopathy, atrial fibrillation, and tachyarrhythmia.<br>**Hematological**: absent expression of the Kx erythrocyte antigen and weakened expression of Kell blood group antigens: hemolysis.<br>RBC acanthocytosis	(17)
			CNV				x		del/dup analyses<br>(40%),<br><br>CMA<br>(30%)				
NKX2-1<br>*(also known as* ***TTF-1****)*	**Benign Hereditary Chorea**	AD (rarer AR)	CNV<br>(2%)				x		del/dup analyses<br>(16%),<br>CMA	Infancy - Childhood	Allelic disorder to:<br>Choreoathetosis, hypothyroidism, and neonatal respiratory distress; Nonmedullary thyroid cancer.<br>Splicing mutations in introns.	Chorea which peaks in the second decade and does not progress. Possible dysarthria, gait abnormalities, slightly decreased intelligence.	(18)
			SNV, del/dup, splice site<br>(82%)	x		x	x						
PDE10A	**PDE10A-related chorea**	AR	SNV	x		x	x			Infancy		Infantile onset chorea, developmental and cognition delay with **normal brain MRI**	(19)
	**PDE10A-related chorea with STRIATAL DEGENERATION**	AD/De novo	SNV	x		x	x			Childhood		Childhood onset chorea, normal cognition and development, with **T2-hyperintense symmetrical bilateral striatal lesions**	(20)
ADCY5	**Dyskinesia with orofacial involvement**	AD (rarer AR) / often De novo	SNV, del, splice site<br>(100%)	x		x	x			Infancy to late-adolescenc e	**Somatic mosaicism** occurring in later stages of embryogenesis, demonstrated in high proportion, in part responsible for intra-familial phenotypic variability.<br>Penetrance 100%.	Dyskinesias: chorea, dystonia, myoclonus, in variable association; ++ face, + arms; constant or paroxysmal, in sleep; typical improvement. in early adulthood.<br>Triggers: none or emotional stress, intercurrent illness, sneezing, caffeine.<br>Sleep related motor and behavior disorder. Alternating hemiplegia of childhood.<br>Possible axial hypotonia, delayed	(21)

												milestones, +/ MCI (often misdiagnosed as CP).	
GPR88	**Chorea, childhood-onset, with psychomotor retardation**	AR	SNV	x		x	x			Infancy		Delayed psychomotor development, Generalized chorea.	(22)
ATM	see Supplementary Table 3												
GNAO1	see Supplementary Table 2												
HPRT1	see Supplementary Table 2												

Supplementary Table 1. Most relevant genetic causes of choreic syndromes and related test selection. For each listed gene, disease nomenclature, reported genetic variants are listed, appropriate diagnostic test selection (based on described genetic variants), important genetic highlights, and main clinical traits are reported. Percentage values reported represent the amount of pathogenetic variants detected with the specific analysis or gene-targeted deletion/duplication analysis on GeneReviews® when available/applicable. **AD**: autosomal dominant. **AOO**: age of onset. **AR**: autosomal recessive. **C**: cytosine. **CMA**: Chromosomal microarray analysis. **CNV**: deletion/insertion/duplication of > 50 base pairs. **del/ins/dup**: deletion/insertion/duplication < 50 base pairs. **del/dup analyses**: gene-targeted deletion/duplication analysis (methods used may include a range of techniques such as quantitative PCR, long-range PCR (**LR-PCR**), multiplex ligation-dependent probe amplification (**MLPA**), and a gene-targeted microarray designed to detect single-exon deletions or duplications). **G**: guanine. **Her**: inheritance. **Expansion**: repeat expansion tests (method used may include long-range PCR (**LR-PCR**), repeat-primed PCR (**RP-PCR**). **LRS**: long-reads sequencing. **mtDNA**: mitochondrial DNA tests; **NGS**: next generation sequencing. **Rpt**: repeats. **SNV**: single nucleotide variant. **splice site**: splice site variant. **T**: thymidine. **UNK**: no data available. **WES**: whole exomes sequencing. **WGS**: whole genome sequencing.

DYSTONIA

Gene	Condition	Her	Variants	NGS panel	Expansion	WES	WGS	mtDNA	Other	AOO	Genetic tricks	Clinical traits	Ref
**Isolated dystonia**													
ANO3	**DYT-ANO3**	AD (rarely de novo)	SNV, splice site	x		x	x			Anytime (peak in adulthood)	Often de novo variants are found in childhood onset.<br>0.8% missense variants in HC (critical evaluation of each variant before establishing causality)	Multifocal cranio-cervical dystonia, often with laryngeal involvement and ULs tremor. Rarely can present with dystonia plus myoclonus; paroxysmal-dystonia.	(23–25)
GNAL	**DYT-GNAL**	AD	SNV, ins (100%)	x		x	x			Adulthood	Incomplete penetrance.<br>Intrafamilial variability.<br>AR in one family, with ID and generalized dystonia	Focal/multifocal cervical dystonia, spreading to face or arms. Dystonic tremor may precede dystonia onset. Rarely can present with dystonia plus myoclonus.	(26–28)
HPCA	**DYT-HPCA**	AR	SNV, del	x		x	x			Childhood - adolescence		Generalized dystonia, starting in limbs, later involving cranio-cervical region. +/- mild syndromic features (ID, seizure, psychiatric features)	(29,30)
KMT2B	**DYT-KMT2B**	AD (mostly de novo)	SNV, del/dupl, splice site (72%)	x		x	x			Childhood; rarely adulthood	Second most common cause of early onset generalized dystonia. Incomplete penetrance. Variable expression	Generalized dystonia starting in limbs, then involving cranial, cervical and laryngeal regions. Commonly reported mild syndromic features (ID, short stature, mild dysmorphic features etc.)	(31,32)
			Chromosomal microdel involving the whole gene (28%)				x		del/dup analyses: CMA				
PRKRA	**DYT-PRKRA**	AR	SNV, del	x		x	x			Childhood - adolescence		Dystonia with prominent oromandibular involvement, dysphagia, retrocollis. +/- mild Parkinsonian features	(33)
THAP1	**DYT-THAP1**	AD	SNV, del/ins	x		x	x			Adolescence	Incomplete Penetrance 50%. One family with large del and severe and atypical form of dystonia.	Cranial or generalized, with prominent tongue and laryngeal involvement	(34)f
TOR1A	**DYT-TOR1A**	AD (rarely de novo)	Mostly 3-bp del in exon 5 (c.907_909delGAG).<br>Exonic SNV (>99%)	x		x	x			Childhood – adolescence; rarely adulthood	First cause of hereditary generalized dystonia (especially in AJ). Incomplete penetrance 35% (modified by the D216H polymorphism). Intrafamilial variability	Generalized dystonia, starting with actions in one limb, then progressively presenting at rest and spreading to other body parts.	(35–39)
VPS16	**DYT-VPS16**	AD, AR	SNV, dupl/del/ins, splice site	x		x	x			Childhood - adolescence	Incomplete penetrance.	Generalized dystonia starting in oromandibular, bulbar/cervical areas or in ULs, slowly progressively.<br>+/- chorea, myoclonus, freezing, ID	(40–43)
EIF2AK2	**DYT-EIF2AK2**	AD<br>(AR 1 case)	SNV	x		x	x			Childhood - adolescence	Incomplete penetrance. Variable expression.<br>Allelic disorder to the Leukoencephalopathy, DD, and episodic neurologic regression	Focal/generalized dystonia. Slowly progressive, may result in gait difficulties, dysarthria/dysphagia.<br>+/- mild DD, pyramidal signs	33236446

											syndrome (AD)		
AOPEP[+]	**DYT-AOPEP**	AR	SNV, del, splice site	x		x	x			Childhood - early adulthood		Multifocal/generalized dystonia, with prominent orofacial, laryngeal involvement. Rarely associated with parkinsonism.	(44,45)
**Combined dystonias (disorders where dystonia frequently coexists with other movement disorders)**													
ATP1A3	**DYT/PARK-ATP1A3** **Rapid-onset Dystonia-Parkinsonism (RDP)**	AD (also de novo)	SNV, ins/del (26%: c.1838C>T) (80-90%)	x		x	x		del/dup analyses	Childhood to adulthood (4-45yo)	Incomplete penetrance. Allelic disorder to: ACH and CAPOS (AD). Variable expression: from only few features of one phenotype to intermediate overlapping phenotypes (+ACH and RPD).	Asymmetric dystonia-parkinsonism with abrupt onset (days-weeks), with later stabilization and slow progression. Cranio-caudal gradient: face/bulbar > UL > LL. Trigger: fever, trauma, exertion. +/- epilepsy, arrhythmia	(46)
TAF1	**DYT/PARK- TAF1** **Lubag syndrome**	XL-R	5 disease-specific changes: 4 SNP and a 48-bp (22); SVA retrotransposon ins (100%)	x		x	x			Adulthood	Only in Filipino descent. Female carriers are mostly asymptomatic, a small minority may have dystonia, parkinsonism or chorea (likely for an extreme X inactivation)	Dystonia-parkinsonism, typically starting with mild parkinsonism, then worsening to a multifocal/generalized dystonia. Rarely pure mild parkinsonism.	(47,48)
GCH1	**DYT/PARK-GCH1** **Dominant DRD / Segawa syndrome**	AD	SNV, ins/del splice site (87%)	x		x	x			Childhood	Penetrance females 87%, males 38%. Variable expression**:** from subtle to severe phenotype	Dystonia-parkinsonism: dystonia, typically starting in LL (equinovarus foot) and gradually progressing to generalized, with later parkinsonism, often pyramidal signs. Diurnal fluctuation. Responsive to low dose L-Dopa.	(49–51)
			large del of multiexons - whole gene (CNV) (13%)				x		del/dup analyses				
TH	**DYT/PARK-TH** **Recessive DRD / Segawa syndrome**	AR	SNV, del, splice site (99%)	x		x	x			Childhood - adolescence	Variable expression**:** from mild to severe phenotype	Broad spectrum: DRD (mild), infantile parkinsonism with DD (severe), infantile encephalopathy (very severe). Rarely can present with myoclonus-dystonia	(52–54)
			Large del (1-2 exons, promotor-exon 1) (1%)				x		MLPA				
DNAJC12	**DYT-DNAJC12** **Hyperphenylalaninemia, mild**	AR	SNV, small del, splice site	x		x	x			Infantile - adulthood	Variable expression (in homozygous twins)	Dystonia-parkinsonism: DD, ID, infantile dystonia, young-onset Dopa-responsive parkinsonism. ↑ **serum phenylalanine** (NBS). Treatment: L-Dopa, 5-HTP, BH4	(55)
			CNV del (up to 6.9 kb)				x		MLPA, CMA				
SLC39A14	**DYT-SLC39A14** **Hypermanganesemia with dystonia-2**	AR	SNV, del, splice site (100%)	x		x	x			Infantile - childhood		Dystonia-parkinsonism: DD, spasticity, bulbar dysfunction, severe generalized dystonia, parkinsonism. **Brain MRI:** globus pallidus T_1 hyper- / T_2 hypo-intensity; ↑↑↑ manganesemia	(56)
KCTD17	**MYC/DYT-KCTD17**	AD	SNV, splice site	x		x	x			Childhood - adolescence		Dystonia with myoclonus: early onset myoclonus, later in life generalized dystonia (+ cranial/cervical). Rarely (2 family): DD, ID, severe lingual dystonia	(57)
SGCE	see Supplementary Table 4											Myoclonus-dystonia.	

GNAO1	**DYT/CHOR-GNAO1**	AD (typically de novo)	SNV, splice site, del (98.5%)	x		x	x			Infancy - adulthood	Variable expression**,** from mild to severe	Broad spectrum: Dystonia (from mild to *status dystonicus*), choreoathetosis, persistent or paroxysmal. +/- DD/ID, epilepsy	(58–60)
			CNV del (intragenic, involving the whole gene or contiguous genes) (1.5%)				x		del/dup analyses: MLPA, CMA				
COX20 (FAM36A)	**DYT-COX20 - Deficiency of cytochrome c oxidase 20**	AR	SNV, splice site	x		x	x			Infancy - childhood	No clear genotype/phenotype correlation	DYTCA: predominant dystonia and mild cerebellar ataxia. Severe forms: DD	(61,62)
**Complex dystonias** (dystonia dominates the clinical picture but occurs in the context of a complex phenotype including symptoms other than movement disorders) and													
DCAF17, PANK2, PLA2G6, CP; FA2H FTL, c19orf12 WDR45 *	**NBIA**	AR, AD (FTL), AD/AR (c19orf12), XL-D (WDR45)	SNV, del/ins/dupl, splice site	x		x	x			Infancy - adulthood	Intrafamilial variability	Dystonia +/- other movement disorders, DD, psychiatric features. Other signs: ataxia, hearing loss, retinal degeneration, hypogonadism, diabetes. **Brain MRI:** basal ganglia T_1 hyperintensity; atrophy (cortex, cerebellum)	(63)
			CNV (del single exons-multiple continuous genes)				x		del/dup analyses: MLPA, CMA				
ATP7B	**Wilson disease**	AR	SNV, del/ins, splice site (98%)	x		x	x			Childhood - elderly (70 yo)	Targeted analysis can be performed first in individuals from populations with known founder variants AJ, Canary Islands, Druze, Sardinia.	Combination of hepatic (fatty liver), psychiatric or neurological signs (movement disorders, ataxia, dysarthria, dementia) **Brain MRI:** putamina T2 hyperintensity ↓ serum ceruloplasmin & copper, ↑ 24-hr urinary copper	(64)
			rare exon/multiexons del/dupl				x		del/dup analyses: MLPA, LRS, long range PCR				
SLC30A10	**Hypermanganesemia with dystonia-1**	AR	SNV, small del/ins (96%)	x		x	x			Childhood - adulthood		Childhood: four-limb dystonia, "cock-walk gait", dysarthria, tremor, bradykinesia. Adulthood: parkinsonism (L-Dopa unresponsive). Other signs: polycythemia, hepatomegaly. **Brain MRI:** basal ganglia, subthalamic /dentate n. T_1 hyperintensity (thalamus and pons spared); ↑↑↑ manganesemia	(65–67)
			Large del (multiple exons) (4%)				x		del/dup analyses: MLPA, LRS, long range PCR				
MECR, SUCLA, ACAT1, OPA1, TIMM8A, mt-ND6	**Mitochondrial disorders**	AR, AD/AR (OPA1), XL-R (TIMM8A), mtDNA	SNV, del, splice site	x		x	x			Infancy - childhood		Various movement disorders (+dystonia), psychiatric abnormalities, ID, seizures, migraine, myopathy, neuropathy, optic/hearing loss. Dysmorphic features, cardiomyopathy, metabolic/endocrine disorders. MELAS, LHON, Leigh syndrome.	(68)i
			CNV (multi-exons/genes del)				x		del/dup analyses: MLPA, CMA				
			SNV mt-DNA					x			Maternal inheritance. Incomplete penetrance, phenotypic variability due to heteroplasmy.		
PTS, MUT, SERAC1, GCDH, PCCA/PCCB; DDC,	**Inborn error of metabolism of aminoacids and monoamine neurotransmitter**	AR	SNV, del/ins, splice site	x		x	x		x	Infancy - childhood		Various movement disorders (+ dystonia), DD/ID, psychiatric abnormalities, seizures. Systemic signs. Often detectable with NBS.	(69,70)

QDPR, SPR												**SPR "dopa responsive dystonia"**	
SLC6A3	**Dopamine Transporter Deficiency Syndrome**	AR	SNV, small del, splice site (95%)	x		x	x			Infancy (classic) - childhood --> adulthood (atypical later onset)	Protection against nicotine dependence	Typical: DD, hyperkinesia or parkinsonism, bulbar and eye movements abnormalities (including oculogyric crises). Atypical later onset: ADHD, tremor, parkinsonism-dystonia, dysarthria.	(71)
			CNV (multiexon del), translocation e ncompassing SLC6A3				x		del/dup analyses: MLPA, CMA				
SLC19A3	**Biotin-responsive basal ganglia disease (BTBGD)**	AR	SNV, del/dup, splice site (95%)	x		x	x			Childhood - adulthood	Biallelic predicted loss-of-function variants are more likely to present early and develop into the severe Leigh-like phenotype. Compound heterozygosity for one missense variant and one predicted loss-of-function variant has been associated with the classic childhood form of BTBGD	Dystonia, Parkinsonism. Classic form in childhood: subacute encephalopathy/coma (often triggered by febrile illness), cranial nerve palsy, seizures. Infancy: Leigh-like syndrome. Adulthood: Wernicke-like encephalopathy. Responsive to thiamine.	(72,73)
			45-kb del in 5'-UTR				x		del/dup analyses: MLPA, CMA				
TUBB4A	**TUBB4A-DYT**	AD	SNV (100%)	x		x	x			Early childhood - early adulthood	Variants consistently associated with phenotypes. Penetrance 100%. Variable clinical and radiological expression (MRI: hypomyelination with atrophy of the basal ganglia and cerebellum or isolated hypomyelination).	Isolated dystonia. Whispering dysphonia, spreading to the neck or limbs, +/- ataxic gait. MRI is normal.	**(74,75)**
	**TUBB4A-Related Leukodystrophy**	AD de novo										Hypomyelinating leukodystrophy-6: pyramidal and extrapyramidal signs (dystonia, perioral dyskinesia, choreoathetosis, oculogyric crisis), cerebellar and bulbar dysfunction, altered cognition. **Brain MRI:** Hypomyelination with atrophy of basal ganglia and cerebellum	
VPS11	**VPS11-related dystonia**	AR	SNV, CNV, splice site	x		x	x		del/dup analyses: MLPA, CMA	Childhood - adolescence	Part of HOPS complex. Biallelic LOF variants.	Complex dystonia with neurodevelopmental features. May present with dystonia, myoclonus, spasticity, cerebral atrophy, neuropathy and overlap with lysosomal storage phenotypes	42
VAC14	**Striatonigral degeneration**	AR	SNV, splice site	x		x	x			Childhood		Striatonigral degeneration, dystonia-parkinsonism, ataxia, dysarthria, hypotonia	(76)
ADAR1	**Aicardi-Goutieres syndrome (AGS)**	AR	SNV, del (100%)	x		x	x			Infancy - early adulthood	A dominant p.Gly1007Arg variant in AGS has been reported	Encephalopathy often with dystonia, DD/ID, intermittent sterile pyrexias	(77,78)
	**Dyschromatosis symmetrica hereditaria**	AD										Dystonia, ID. Hyper-/Hypo-pigmented macules on face and extremities	(79)
IRF2BPL	**Neurodevelopmental disorder with regression, abnormal movements, loss of speech, and seizures**	AD (de novo)	SNV, del/ins	x		x	x				Intronless gene	DD, hypotonia, seizures, pyramidal signs, dysarthria	(80)
FOXG1	**FOXG1 Syndrome**	AD (also de novo)	SNP, del (>95%)	x		x	x			Childhood	Del/intragenic pathogenic variants vs large del/dup (whole	Rett-like phenotype: DD, ID, hyperkinetic movements,	(81–83)

			CNV (large del, whole gene dup) (<5%)				x		del/dup analyses: MLPA		gene / contiguous gene): different phenotypes. Reported mosaicism.	stereotypies, psychiatric manifestations, impairment of social interaction	
HPRT	**Lesch-Nyhan syndrome**	XL	SNP, splice site, del/ins/dup (80%)	x		x	x			Childhood	Heterozygous females are clinically normal; rarely LND is observed for skewed X-chromosome inactivation	Dystonia, chorea, occasionally ballism, DD/ID, eye movement abnormalities, spasticity, compulsive self-injurious behavior (resembling cerebral palsy). Additional clinical features: hyperuricemia, crystalluria, gouty arthritis, nephrolithiasis, renal failure. **↓↓↓ HGprt enzyme activity**	(84,85)
			CNV (5'UTR, one exon, multiexons) (20%)				x		del/dup analyses: MLPA, CMA				
GLB1	**GM1 gangliosidosis** (lysosomal storage disorder)	AR	SNV, del, intronic SNV (>99%)	x		x	x			Infancy - adulthood	Variable expression: type I, earlier onset and more severe phenotype. Type III: childhood to III decade.	Type III: generalized dystonia, gait and speech problems, akinetic rigid parkinsonism, cardiomyopathy, cognitive decline. **↓ beta-galactosidase enzyme activity**	(86)
			CNV (del) (<1%)						del/dup analyses: MLPA				
SQSTM1	**Neurodegeneration with ataxia, dystonia, and gaze palsy (NADGP)**	AR	SNV, del/ins, splice site	x		x	x				Allelic disorder to Frontotemporal Dementia and/or Amyotrophic Lateral Sclerosis 3	Childhood-onset neurodegeneration, gait ataxia, cognitive decline, oculomotor abnormalities including vertical gaze palsy and nystagmus, and hypergonadotropic hypogonadism	(87)
ACTB	**Dystonia-deafness**	AD (mainly de novo)	SNV (100%)	x		x	x			Adolescence - adulthood		Sensory hearing loss, generalized dystonia, skeletal abnormalities. +/- DD, seizures	
BCAP31		XL-R	SNV, splice site	x		x	x			Infancy		Sensorineural hearing loss. generalized dystonia. Central hypomyelination, ID, microcephaly, ophthalmoplegia	(88)
			CNV (del 5.3 kb)				x		del/dup analyses: MLPA, CMA				
FITM2		AR	SNP, dup	x		x	x			Childhood		Sensorineural hearing loss. Generalized dystonia. Other signs: DD, poor growth	(89)
ATXN3*	**SCA-3** (see Supplementary Table 3)	AD	exonic CAG expansion		x		x		LRS	Adult	Penetrance depending on number of expansions. Anticipation, especially with paternal transmission.	May have dystonia, parkinsonism >> ataxia. Altered eye movements (+horizontal), lower motoneuron involvement, neuropathy	(90)
KIF1C*	**HSP/ATX-KIF1C** **SPASTIC ATAXIA 2**	AR	SNP, splice site	x		x	x			Adulthood		Pure and complicated; variable additional features including dystonia, ataxia, chorea, myoclonus, dysarthria, developmental delay, mild mental retardation, hypodontia, ptosis, short stature, sensorineural deafness, pes planus, white matter lesions	(91)
			CNV (del multiexons)				x		del/dup analyse: MLPA, CMA				
DNAJC6*	**PARK-DNAJC6**	AR	SNP, del splice site (95%)	x		x	x			Early adulthood (<21 years)		Atypical parkinsonism: DD/ID, parkinsonism, dystonia, spasticity, seizures, bulbar/gastrointestinal disfunction	(92,93)
			CNV (del 80 kb) (5%)				x		del/dup analyse: MLPA,				

									CMA				
**Other candidate genes associated with dystonia**													
NUP54* *(2023)	**Dystonia with striatonigral degeneration**	AR	SNV, del	x		x	x			Childhood - adolescence		Generalized dystonia, DD, limb-choreoathetoid and/or ataxic movements, dysarthria, dysphagia. **Brain MRI:** T2/FLAIR hyperintensities in basal ganglia	(94)
NUP62* *(2006)	**Dystonia with striatonigral degeneration**	AR	SNV	x		x	x			Infancy		Choreoathetosis, dystonia, and abnormal high T2-weighted MRI signals in the striatum	(95)
DRD1* *(2023)	**-**	AR	SNV	x		x	x			Infancy		Severe infantile parkinsonism-dystonia, oculogyric crises, dysautonomia. severe DD	(96)
DRD2* *(2021)	**-**	AD	SNV	x		x	x			Childhood - Early adulthood		Focal or multifocal Dystonia, generalized chorea. Psychiatric symptoms	(97)
ATP5F1B* *(2023)	**-**	AD	SNV (2 families)	x		x	x			Infancy - adolescence	Encoding a subunit of the mitochondrial ATP synthase	Early-onset isolated dystonia	(98)

Supplementary Table 2. Most relevant genetic causes of dystonic syndromes and related test selection. For each listed gene, disease nomenclature, reported genetic variants are listed, appropriate diagnostic test selection (based on described genetic variants), important genetic highlights, and main clinical traits are reported. Percentage values reported represent the amount of pathogenetic variants detected with the specific analysis or gene-targeted deletion/duplication analysis on GeneReviews® when available/applicable. **A**: adenine. **ACH**: alternating hemiplegia of childhood; **AD**: autosomal dominant; **AJ**: Ashkenazi Jews; **AOO**: age of onset. **AR**: autosomal recessive; **C**: cytosine. **CAPOS**: cerebellar ataxia, areflexia, pes cavus, optic atrophy, and sensorineural hearing loss. **CMA**: Chromosomal microarray analysis. **CNV**: deletion/insertion/duplication of > 50 base pairs. **DD**: developmental delay. **del/ins/dup**: deletion/insertion/duplication < 50 base pairs. **del/dup analyses**: gene-targeted deletion/duplication analysis (methods used may include a range of techniques such as quantitative PCR, long-range PCR (**LR-PCR**), multiplex ligation-dependent probe amplification (**MLPA**), and a gene-targeted microarray designed to detect single-exon deletions or duplications). **DRD**: Dopa responsive dystonia. **Expansion**: repeat expansion tests (method used may include long-range PCR (**LR-PCR**), repeat-primed PCR (**RP-PCR**). **G**: guanine. **HC**: healthy controls. **Her**: inheritance. **ID**: intellectual disability. **LHON**: Leber hereditary optic neuropathy. **LL**: lower limb. **MELAS**: Mitochondrial encephalomyopathy, lactic acidosis and stroke-like episodes. **mtDNA**: mitochondrial DNA tests. **NBS**: new born screening. **NGS**: next generation sequencing. **Rpt**: repeats. **SNV**: single nucleotide variant. **Splice site**: splice site variant. **SVA**: SINE-VNTR-Alu. **T**: thymidine. **UL**: upper limb. **UNK**: no data available. **WES**: whole exomes sequencing. **WGS**: whole genome sequencing. *****: Disorders that usually present with other phenotypes but can have predominant dystonia.

ATAXIA

Gene	Condition	Her	Variants	NGS panel	Expansion	WES	WGS	mtDNA	Other	AOO	Genetic tricks	Clinical traits	Ref
ATXN1, ATXN2, ATXN3, ATXN7, ATXN8, PPP2R2B	SCA1,2,3,7,8, 12	AD	Exonic CAG expansion (SCA12: promoter) (100%)		x		x		LRS	Adults	Anticipation (>> with parental transmission)	Different additional features in different subtypes (neuropathy: SCA1; slow horizontal saccades: SCA2; parkinsonism and dysautonomia: SCA1,2,3; spasticity: SCA3; prominent tremor: SCA12)	(99,100)
ATXN10	SCA10	AD	Intronic ATTCT expansion (100%)		x		x		LRS	Adults	Anticipation (>> with parental transmission), Mexican and Brasilian	Cerebellar symptoms and seizure	(101)
TBP	SCA17	AD	Exonic CAG or CAA expansion (100%)		x		x		LRS	Adults	Anticipation (>> with parental transmission)	Great mimic: chorea, motoneuron disease, cognitive impairment	(102)
NOP56	SCA36	AD	Intronic GGCCTG expansion (100%)		x		x		LRS	Adults	Anticipation (>> with parental transmission)	Associated with motoneuron symptoms	(103)
BEAN1	SCA31	AD	(TAGAA)n repeat (100%)		x		x		LRS	Adults	Anticipation (>> with parental transmission), common in Japan	Variable presence of hearing loss, common in Japan	(104)
CACNA1A	SCA6, EA2	AD	CAG expansion		x		x		LRS	Childhood/ adolescence		SCA, episodic ataxia type 2 (prolonged attacks of cerebellar symptoms and blurred vision)	(105)
			SNV	x		x	x						
KCNA1, CACNB4, SLC1A3, SCN2A	EA1, EA5, EA6, EA9	AD	SNV, in/del	x		x	x			Childhood/ adolescence		Brief (EA1,EA9) or prolonged (EA5,EA6,EA9) attacks of cerebellar symptoms, dizziness, blurred vision, nausea/vomiting, possible epilepsy	(106)
RFC1	CANVAS	AR	Intronic expansions (100%)		x		x		LRS	Adults		Neuropathy, vestibular areflexia	(107)
FGF14	SCA27B	AR	GAA Intronic expansions (100%)		x		x		LRS	Adults		Downbeat ny, episodic symptoms	(108,109)
ATN1	DRPLA	AD	CAG expansion (100%)		x		x		LRS	Childhood to adulthood	Age and CAG expansion correlation (<21 y: 63-79; 21-40y: 61-69; >40y: 48-67)	Early onset: seizure, ataxia, myoclonus, ID Late onset (>20y): ataxia, choreoatetosis, psychiatric symptoms and dementia	(110,111)
FMR1	FXTAS	X-linked	CGG repeats (100%)		x		x		LRS	Adults	Premutation alleles (55-200	Changes in personality and psychiatric	(112)

											repeats)	symptoms. Women: rare (with POCS)	
**Mitochondrial DNA**	NARP, MERRF, KSS, Leigh syndrome							x			Maternal inheritance	Myopathy, neuropathy, hearing loss, optic atrophy, dementia, and short stature, myopathy, neuropathy, hearing loss, optic atrophy, dementia, and short stature	(113)
TTPA	AVED	AR	SNV (>97%)	x		x	x			I-II decade	One case reported of whole gene deletion	Cerebellar features plus areflexia and loss of proprioception, Babinski sign, macular degeneration and pigmentary retinopathy. Blood work: low levels of vitamin E	(114,115)
			SVs/CNV	x			x		del/dup analyses: MLPA, LRS, long range PCR				
APOB, MTTP	Ataxia and vitamin E deficiency	AR	SNV, in/del	x		x	x			I-II decade	*MTTP*: small indels more frequent than missense variants	Neuropathy, retinopathy, acanthocytosis, hepatomegaly, deficiency of fat-soluble vitamins	(116)
FXN	Friedreich ataxia (FRDA)	AR	GAA repeats (96%)		x		x		LRS	Usually < 25y (25-39y: LOFA; >40y: VLOFA)	5-33 GAA: normal; 34-65: premutation; >66: full penetrance	Reduced reflexes, positive Babinski sign; hypertrophic cardiomyopathy; DM; hypoacusia. Late onset forms can have present reflexes	(117–120)
			SNV	x		x	x			4% cases (mostly compound heterozygous with expansion of the other allele)			
			SVs/CNV				x		del/dup analyses: MLPA, LRS, long range PCR	Rare cases			
APTX, SETX, ATM	AOA	AR	SNV (90-95% *ATM*, 80-92% *SETX*)	x		x	x		LRS	Childhood to adulthood	*ATM*: deep intronic variants reported	Oculomotor apraxia, dystonia, increased AFP (*ATM*), hypoalbuminemia (*APTX*), increased cancer risk (*ATM*)	(121–125)
			SVs/CNV (5-10% *ATM*, 8-20% *SETX*)	x		x	x		del/dup analyses: MLPA, LRS, long range PCR				
SACS	Charlevoix-Saguenay	AR	SNV (95%)	x	x	x				Adults	Frequent in French Canadians	Ataxia and spasticity	(126)
			CNV (5%)				x		del/dup analyses:				

									MLPA, LRS, long range PCR, CMA				
Paraplegin	SPG7	AR,	SNV (>98%)	x		x	x			Adults		Ataxia and spasticity	(99)
			SVs/CNV (2%)	x		x	x		del/dup analyses: MLPA, LRS, long range PCR, CMA				
ANO10	SCAR10	AR	SNV, in/del (100%)	x		x	x			III-V decade		Reported spasticity, neuropathy, reduced levels of CoQ10	(127,128)
SYNE1	SCAR8	AR	SNV (~100%)	x		x	x			Childhood to adulthood	Multiple isoforms with variable tissue expression (Variants causing SCAR8 involve CNS isoform); frequent intronic variants affecting the splicing	Hotspot in Quebec, Canada (where it manifests as pure, slow progressive cerebellar ataxia). Other cases are associated with spasticity and brainstem dysfunction.<br><br>Allelic with Arthrogryposis multiplex congenita 3 and Emery-Dreifuss muscular dystrophy 4	(129–132)
			CNV				x		del/dup analyses: MLPA, LRS, long range PCR, CMA				
ITPR1	SCA15 and 29	AD	SNV	x		x	x			Adult (SCA15) and childhood (SCA29)	Allelic with Gillespie syndrome (AD/AR)	SCA15: adult-onset, pure ataxia, slowly progressive; SCA29: non-progressive, infantile onset, with possible cognitive impairment	(133,134)
			SVs/CNV				x		del/dup analyses: MLPA, LRS, long range PCR				
KCND3	SCA19/22	AD	SNV, in/del	x		x	x			Childhood to adulthood	Loss of function pathogenic variants are associated with SCA19/22, gain of function pathogenic variants are associated with Brugada syndrome	Ataxia with possible cognitive deficit/developmental delay, other movement disorders	(135,136)
SPTBN2	SCA5 / SCAR14	AD/AR	SNV	x		x	x			I-V decade	Anticipation has been reported	Ataxia, possible cognitive impairment, downbeat nystagmus, spasticity	(137,138)
			SVs/CNV				x		del/dup analyses: MLPA, LRS, long range PCR				

AFG3L2	SCA28/SCAR5	AD/AR	SNV, in/del (>99%)	x		x	x			Childhood to adulthood	Deletions and duplications are very rare	Associated with mitochondrial features (PEO, ptosis) and spasticity	(139)
			CNV				x		del/dup analyses: MLPA, LRS, long range PCR, CMA				
STUB1	SCA48/SCAR16	AD/AR	SNV, in/del	x		x	x			Mid-adulthood (SCA48); I-II decade (SCAR16)		SCA48: ataxia, cognitive impairment, psychiatric symptoms and other movement disorders; SCAR16: ataxia, neuropathy, hyperreflexia, some with gonadal dysfunction and /or cognitive impairment	(140,141)
DAB1	SCA37	AD	ATTTC(n) repeat insertion (100%)		x				LRS, PCR, Southern blot	Adult	ATTTC(n) insertion in 5'-UTR of *DAB1* intron 3 or in noncoding regulatory region of *DAB1*	Pure ataxia	(142)
ADCK3	SCAR9	AR	SNV, in/del	x		x	x			Childhood		Primary coenzyme Q10 deficiency-4 (COQ10D4), Ataxia with possible epilepsy and cognitive deficits	(99,143)

Supplementary Table 3. Most relevant genetic causes of ataxia syndromes and related test selection. For each listed gene, disease nomenclature, reported genetic variants are listed, appropriate diagnostic test selection (based on described genetic variants), important genetic highlights, and main clinical traits are reported. Percentage values reported represent the amount of pathogenetic variants detected with the specific analysis or gene-targeted deletion/duplication analysis on GeneReviews® when available/applicable. **AD**: autosomal dominant; **AFP**: alpha fetoprotein; **AOA**: ataxia with oculomotor apraxia; **AVED**: ataxia with vitamin E deficiency; **AR**: autosomal recessive; **CMA**: Chromosomal microarray analysis**. CNV**: deletion/insertion/duplication of > 50 base pairs; **del/ins/dup**: deletion/insertion/duplication < 50 base pairs. **del/dup analyses**: gene-targeted deletion/duplication analysis (methods used may include a range of techniques such as quantitative PCR, long-range PCR (**LR-PCR**), multiplex ligation-dependent probe amplification (**MLPA**), and a gene-targeted microarray designed to detect single-exon deletions or duplications); DM: diabetes mellitus; **FXTAS**: Fragile X-associated tremor/ataxia syndrome; **KSS**: Kearns-Sayre syndrome; **LRS**: long read sequencing; **MERRF**: myoclonic epilepsy with ragged-red fibers; **NARP**: Neuropathy, Ataxia, Retinitis Pigmentosa; **NGS**: next generation sequencing; **PEO**: progressive external ophthalmoplegia; **POCS**: premature ovarian failure; **SCA**: spinocerebellar ataxia; **SCAR**: spinocerebellar ataxia recessive; **SNV**: single nucleotide variant; SVs: structural variants; **WES**: whole exomes sequencing**. WGS**: whole genome sequencing.

MYOCLONUS

Gene	Condition	Her	Variants	NGS panel	Expansion	WES	WGS	mtDNA	Other	AOO	Genetic tricks	Clinical traits	Ref
CSTB	Unverricht-Lundborg disease (EPM1)	AR	CCC-CGC-CCC-GCG expansion (90%)		x		x		LRS	Late childhood or adolescence	2-3 repeats: normal;<br>12-17 repeats: uncertain: ≥ 30 repeats: full penetrance (4-11 and 18-29 not been observed). High incidence in Finland and North Africa	Frequent generalized seizures + cerebellar signs +/- cognitive decline/dementia<br><br>Compound heterozygous may cause more severe phenotype, mostly in makes	(144–148)
			SNV (10%)	x		x	x						
Laforin (EPM2A), NHLRC1 (Malin, EPM2B)	Lafora disease (EPM2)	AR	SNV (85%-90% EPM2A, >90% *NHLRC1*)	x		x	x			Adolescence		Lafora disease: occipital and generalized seizures, Lafora bodies (polyglucosans or glycogen) on skin biopsy	(149,150)
			SVs/CNV (10-15% EPM2A, <10% *NHLRC1*)				x		del/dup analyses: MLPA, LRS, long range PCR				
MT-TK (90%)<br>MT-TF, -TH, -TI, -TL1, -TP, TS1, -TS2	Myoclonic epilepsy with ragged-red fibers (MERRF)	MT	SNV					x		Childhood to adulthood	Consider testing different tissues to account for heteroplasmy	Myopathy, neuropathy, hearing loss, optic atrophy,<br>dementia, and short stature, myopathy, neuropathy,<br>hearing loss, optic atrophy,<br>dementia, and short stature	(151,152)
ATN1	DRPLA	AD	CAG expansion (100%)		x		x		LRS	Childhood to adulthood	Age and CAG expansion correlation (<21 y: 63-79; 21-40y: 61-69; >40y: 48-67)	Early onset: seizure, ataxia, myoclonus, ID<br>Late onset (>20y): ataxia, choreoatetosis, psychiatric symptoms and dementia	(111,153)
NEU1	Sialidosis type 1	AR	SNV	x		x	x			II-III decade		Cherry red spot myoclonus (+ ataxia), Neuroaminidase activity on leucocytes	(154)
GBA	Gaucher disease type 3	AR	SNV (99%)	x		x	x						
			SV (complex rearrangement *GBA-GBAP1*) (1%)						Targeted gene sequencing for complex arrangements and del/dup analyses: MLPA, LRS, long range	I decade	*GBAP1* (pseudogene) has >96% homology with *GBA1*	Oculomotor apraxia (mostly horizontal gaze), systemic symptoms (hepatomegaly, splenomegaly, bone disease, cytopenia, pulmonary disease)	(155)

									PCR, CMA				
SCARB2/ LIMP2	Action myoclonus renal failure syndrome (EPM4)	AR	SNV (100%)	x		x	x			Childhood to adulthood		Renal failure due to steroid-resistant nephrotic syndrome (not present in 100% cases)	(156,157)
PRICKLE1	PRICKLE1-PME with ataxia (EPM5)	AR (AD)	SNV (100%)	x		x	x			I decade		Heterozygous patients with epilepsy, autism, developmental delay and brain malformation have been reported	(158–162)
GOSR2	North see PME with ataxia (EPM6)	AR	SNV, in/del	x		x	x			< 1 year	G144W most frequent variant	Loss of ambulation in the II decade; cognition may be initially spared	(163)
ASAH1	SMA-PME	AR	SNV (92%)	x		x	x			Childhood	Allelic with Farber disease (neonatal onset of painful joint deformity, subcutaneous nodules, granulomas of larynx and epiglottis, life expectancy <2 years)	Proximal weakness, respiratory difficulties, possible ID	(164)
			SVs/CNV (8%)	x		x	x		del/dup analyses: MLPA, LRS, long range PCR				
DHDDS	CDG	AR	SNV	x		x	x			Childhood		Variable phenotype, including: ataxia, ID, PME, facial, myoclonus, dystonia, chorea	(165)
NUS1	CDG, PMA	AR, AD	SNV, in/del	x		x	x		del/dup analyses: MLPA, LRS, long range PCR	Childhood		Variable phenotype, including: ataxia, ID, PME, facial, myoclonus	(165–167)
SGCE	Myoclonus-dystonia	AD	SNV, in/del (75%)	x		x	x			Childhood	Paternal inherited due to maternal imprinting (5% imprinting escape)	Shivering myoclonus, Alcohol response, OCD/anxiety<br><br>Silver-Russel syndrome (myoclonus/dystonia + growth retardation and dysmorphism)	(168–174)
			SVs/CNV (25%)	x		x	x		del/dup analyses: MLPA, LRS, long range PCR				
KCTD17, ANO3, ADCY5, ATP1A3, YY1, VPS16	Dystonia with myoclonus	AD/AR	SNV	x	x	x				Childhood to adulthood	SVs (including CNV: microdeletion) reported for *VPS16*	Various degrees of combination of dystonia (mostly upper limbs and neck) and myoclonus (mostly cortical)	(43,175)
			SVs/CNV (*VPS16*)	x		x	x		del/dup analyses: MLPA, LRS, long range PCR				
HTT	Huntington disease	AD	CAG expansion (100%)		x		x			Adulthood > childhood	Anticipation (< 26 CAG expansion: normal; 27-35:	Classic form: chorea, dementia, psychiatric symptoms.<br>Juvenile form Westphal	(176,177)

											intermediate; >46: pathogenic)	variant): rigid-hypokinetic	
**SCA20, *GFAP* , *NF*, *POLG*, *GM2*, *CYP27A1***	PAPT	AD/AR	SNV, in/dell	x		x	x			Adulthood	SCA20: identification of a duplicated region that segregates with the disease but no specific gene has been identified yet	Adult onset of palatal tremor (myoclonus) and associated with ataxia; brain MRI: hyperintensity of the olives. *GFAP*: associated with Alexander disease; POLG: possible mitochondrial features; *NF*: iron accumulation at brain MRI; *GM2*: cherry red spot (retina) and neuropathy; *CYP27A1* associated with CTX: tendon xanthomas, cataracts, diarrhea, neonatal jaundice, brain MRI: T2 hyperintensities of the dentate nuclei, basal ganglia, cerebral peduncles, cerebellar white matter, and periventricular white matter	(63,178–183)
			SVs/CNV (duplication 11q12.2-11q12.3 In SCA20)				x		CMA, MLPA, LRS, long range PCR, FISH				
SAMD12, STARD7, MARCHF6, YEATS2, TNRC6A, RAPGEF3	Cortical tremor	AD	Intronic expansion				X*		RP-PCR, LRS	Adulthood		Family history of cortical myoclonus, epilepsy in at least one family member, AD inheritance	(184,185)
	**Epileptic syndromes**	AD/AR	SNV	x		x	x		Microarray	Childhood			(186)
			SVs/CNV	x		x	x		MLPA, LRS, long range PCR				
			Chromosomal abnormalities						CMA				

Supplementary Table 4. Most relevant genetic causes of myoclonic syndromes and related test selection. For each listed gene, disease nomenclature, reported genetic variants are listed, appropriate diagnostic test selection (based on described genetic variants), important genetic highlights, and main clinical traits are reported. Percentage values reported represent the amount of pathogenetic variants detected with the specific analysis or gene-targeted deletion/duplication analysis on GeneReviews® when available/applicable. *Not available diagnostically. **AD**: autosomal dominant; **AR**: autosomal recessive; **CDG**: congenital disorder of glycosylation; **CMA**: Chromosomal microarray analysis. **CNV**: deletion/insertion/duplication of > 50 base pairs; CTX: Cerebrotendinous Xanthomatosis; **del/ins/dup**: deletion/insertion/duplication < 50 base pairs. **del/dup analyses**: gene-targeted deletion/duplication analysis (methods used may include a range of techniques such as quantitative PCR, long-range PCR (**LR-PCR**), multiplex ligation-dependent probe amplification (**MLPA**), and a gene-targeted microarray designed to detect single-exon deletions or duplications); **ID**: intellectual disability; **EPM1/EPMI2A/EPM4/EPM5**: progressive myoclonic epilepsy, type 1/2A/4/5; **LRS**: long read sequencing; **NGS**: next generation sequencing; PAPT: progressive ataxia with palatal tremor; PMA: progressive myoclonus ataxia; **PME**: progressive myoclonic epilepsy; **SMA-PME**: spinal muscular atrophy-progressive myoclonic epilepsy; **SNV**: single nucleotide variant; **SVs**: structural variants; **WES**: whole exomes sequencing. **WGS**: whole genome sequencing.

PAROXYSMAL MOVEMENT DISORDERS (PMD)

Gene	Condition	Her	Variants	NGS panel	Expansion	WES	WGS	mtDNA	Gene-targeted del/dup analysis	AOO	Genetic tricks	Clinical traits	REF
**Paroxysmal dyskinesia**													
PRRT2	***PRRT2 disorders***	AD	SNV, splice site (>99%)	x		x	x		del/dup analyses (<1%)	Infancy - childhood (epilepsy), adolescence - adulthood (PKD or migraine)	Penetrance 50-90%. Intrafamilial and interfamilial variability. Biallelic variants (<1%): more severe phenotype.	Three core phenotypes (independent or overlapped): 1) Seizures: IC, BFIE; 2) PMD: +PKD, EA (rarely PED, PNKD); 3) Headache: migraine w/ or w/o aura, HM. Between 1-2: ICCA Responsive to CMZ.	(187)
MR1	***PKND***	AD	SNV, del (100%)	x		x	x			Childhood - adolescence (rarely adulthood)	Penetrance 98%	PKND. Triggers: coffee, tea, or alcohol, excitement, stress, or fatigue, or spontaneous	(188)
SLC2A1	***GLUT1 deficiency syndrome***	AD (90% de novo)	SNV, del (84%)	x		x	x				Complete penetrance. Variable expression with two phenotypes.	**Classic** 90%: epilepsy, DD, dysarthria, PEID with complex movement including ataxia, dystonia, chorea. **Non-classic** 10%, milder: PD with ataxia, choreoathetosis, dystonia, alternating hemiplegia. Responsive to ketogenic diet. **CSF glucose concentration <60 mg/dL**	(189,190)
			CNV (multiexon or whole-gene del) (13%)				x		del/dup analyses				
**Episodic ataxia (EA)**													
KCNA1	***EA Type 1***	AD	SNV (>90%)	x		x	x			Childhood - adolescence (<20yo)	Potassium channelopathy	**Brief** attacks (Secs-Mins) with variable symptoms including vertigo, blurred vision, diplopia, nausea, headache, diaphoresis, clumsiness, stiffening, dysarthria, difficulty in breathing. **Interictal** myokymia, episodes of spastic contractions of the muscles with loss of coordination/balance. +/- epilepsy, DD, ID.	(191)
CACNA1A	***EA Type 2***	AD	SNP, del/dup/ins, splice site (95%)	x		x	x			Childhood - adulthood	Phenotypic variability. Allelic disorder to: familial HM; SCA6 (due to repeated CAG expansions). *Progressive cerebellar ataxia +/- migraine*	**Long** attacks (mins-hours-days) **Interictal** nystagmus; +/- chronic ataxia and epilepsy, 50% migraine	(192)
			CNV (7-140 kb, multiexons)				x		del/dup analyses				(192)
			CAG expansion		x		x						(193)
unknown (locus 1q42)	***EA Type 3***	AD	unknown	NA						Childhood - adult		**Brief** attacks (mins), like EA1 plus tinnitus; +/- epilepsy	(194)
unknown (linkage excluded with EA1-2)	***EA Type 4***	AD	unknown	NA						Adulthood		**Long** attacks (hours), like EA2 without nystagmus; +/- epilepsy	(194)
CACNB4	***EA Type 5***	AD	SNV	x		x	x			Adulthood		**Long** attacks (hours), like EA2 **Interictal** nystagmus, epilepsy	(195)
SLC1A3	***EA Type 6***	AD	SNV	x		x	x			Childhood - adulthood		**Long** attacks (hours), like EA2 +/- migraine, alternating hemiplegia	(194)
unknown (Maps 19q13)	***EA Type 7***	AD	unknown	NA						unk		Long attacks (hours), like EA2 without nystagmus	(194)

UBR4	***EA Type 8?***	AD	SNV	x		x	x			Infancy		**Brief and long** attacks (mins-hours), overlaps with EA1 and 2, +/- myokymia or nystagmus, intentional tremor	(196)
FGF14	***EA Type 9?***	AD	SNV, ins	x		x	x			Childhood - adult	Phenotypic variability. Allelic disorder to: SCA27	**Brief and long** attacks (secs-days), like EA2 with tremor	(197)
			GAA expansion		x		x		LRS				
SCN1A		AD	SNV	x		x	x			Infancy - childhood	Allelic disorder to: Dravet syndrome and other epileptics syndromes, familial HM	**Brief and long** attacks (mins-hours), **Interictal** epilepsy, neonatal seizures	(194)
CACNA1G	***EA Type 10? or Episodic vestibulocerebellar ataxias***	AD	SNV	x		x	x			Adulthood	Allelic disorder to: SCA42	**Long** attacks (up to months), **Interictal** chronic ataxia; also: facial numbness, movement induced vertigo, bilateral vestibulopathy	(198)

Supplementary Table 5. Most relevant genetic causes of paroxysmal movement disorders and related test selection. For each listed gene, disease nomenclature, reported genetic variants are listed, appropriate diagnostic test selection (based on described genetic variants), important genetic highlights, and main clinical traits are reported. Percentage values reported represent the amount of pathogenetic variants detected with the specific analysis or gene-targeted deletion/duplication analysis on GeneReviews® when available/applicable. **A**: adenine. **AD**: autosomal dominant. **AOO**: age of onset. **AR**: autosomal recessive. **BFIE**: benign familial infantile epilepsy. **CNV**: deletion/insertion/duplication of > 50 base pairs. **del/ins/dup**: deletion/insertion/duplication < 50 base pairs. **del/dup analyses**: gene-targeted deletion/duplication analysis (methods used may include a range of techniques such as quantitative PCR, long-range PCR (**LR-PCR**), multiplex ligation-dependent probe amplification (**MLPA**), and a gene-targeted microarray designed to detect single-exon deletions or duplications). **EA**: Episodic ataxia. **Expansion**: repeat expansion tests (method used may include long-range PCR (**LR-PCR**), repeat-primed PCR (**RP-PCR**). **G**: guanine. **HM**: Hemiplegic migraine. **Her**: inheritance. **IC**: Infantile convulsions. **ICCA**: Infantile convulsions with paroxysmal choreoathetosis**. LRS**: long-reads sequencing. **mtDNA**: mitochondrial DNA tests; **NGS**: next generation sequencing. **PD**: paroxysmal dyskinesia. **PED**: Paroxismal exercise-induced dyskinesia. **PKD**: Paroxismal kinesigenic dyskinesia. **PNKD**: Paroxismal non kinesigenic dyskinesia. **SNV**: single nucleotide variant. **splice site**: splice site variant. **T**: thymidine. **UNK**: no data available. **WES**: whole exomes sequencing. **WGS**: whole genome sequencing.

References

1. Bean L, Bayrak-Toydemir P. American College of Medical Genetics and Genomics Standards and Guidelines for Clinical Genetics Laboratories, 2014 edition: technical standards and guidelines for Huntington disease. Genet Med. 2014 Dec;16(12):e2.

2. McColgan P, Tabrizi SJ. Huntington's disease: a clinical review. Eur J Neurol. 2018 Jan;25(1):24–34.

3. Mead S. Prion disease genetics. Eur J Hum Genet. 2006 Mar;14(3):273–81.

4. Margolis RL, Holmes SE, Rosenblatt A, Gourley L, O'Hearn E, Ross CA, et al. Huntington's Disease-like 2 (HDL2) in North America and Japan. Ann Neurol. 2004 Nov;56(5):670–4.

5. Margolis RL, O'Hearn E, Rosenblatt A, Willour V, Holmes SE, Franz ML, et al. A disorder similar to Huntington's disease is associated with a novel CAG repeat expansion. Ann Neurol. 2001 Dec;50(6):373–80.

6. Kambouris M, Bohlega S, Al-Tahan A, Meyer BF. Localization of the gene for a novel autosomal recessive neurodegenerative Huntington-like disorder to 4p15.3. Am J Hum Genet. 2000 Feb;66(2):445–52.

7. Magri S, Nanetti L, Gellera C, Sarto E, Rizzo E, Mongelli A, et al. Digenic inheritance of STUB1 variants and TBP polyglutamine expansions explains the incomplete penetrance of SCA17 and SCA48. Genet Med. 2022 Jan;24(1):29–40.

8. Maltecca F, Filla A, Castaldo I, Coppola G, Fragassi NA, Carella M, et al. Intergenerational instability and marked anticipation in SCA-17. Neurology. 2003 Nov 25;61(10):1441–3.

9. Hire RR, Katrak SM, Vaidya S, Radhakrishnan K, Seshadri M. Spinocerebellar ataxia type 17 in Indian patients: two rare cases of homozygous expansions. Clin Genet. 2011 Nov;80(5):472–7.

10. Bauer P, Laccone F, Rolfs A, Wüllner U, Bösch S, Peters H, et al. Trinucleotide repeat expansion in SCA17/TBP in white patients with Huntington's disease-like phenotype. J Med Genet. 2004 Mar;41(3):230–2.

11. Burke JR, Wingfield MS, Lewis KE, Roses AD, Lee JE, Hulette C, et al. The Haw River syndrome: dentatorubropallidoluysian atrophy (DRPLA) in an African-American family. Nat Genet. 1994 Aug;7(4):521–4.

12. Becher MW, Rubinsztein DC, Leggo J, Wagster MV, Stine OC, Ranen NG, et al. Dentatorubral and pallidoluysian atrophy (DRPLA). Clinical and neuropathological findings in genetically confirmed North American and European pedigrees. Mov Disord. 1997 Jul;12(4):519–30.

13. Silvia Prades, Claudio Melo de Gusmao, Silvia Grimaldi, Yael Shiloh-Malawsky, Thomas Felton, and Henry Houlden. DRPLA. 1999 Aug 6 [Updated 2023 Sep 21]. In: GeneReviews® [Internet] [Internet]. Seattle (WA): University of Washington, Seattle; 1993-2024. Margaret P Adam, Jerry Feldman, Ghayda M Mirzaa, Roberta A Pagon, Stephanie E Wallace, Anne Amemiya; Available from: https://www.ncbi.nlm.nih.gov/books/NBK1491/

14. Gijselinck I, Cruts M, Van Broeckhoven C. The Genetics of C9orf72 Expansions. Cold Spring Harb Perspect Med. 2018 Apr 2;8(4):a026757.

15. Dobson-Stone C, Danek A, Rampoldi L, Hardie RJ, Chalmers RM, Wood NW, et al. Mutational spectrum of the CHAC gene in patients with chorea-acanthocytosis. Eur J Hum Genet. 2002 Nov;10(11):773–81.

16. Ueno S, Maruki Y, Nakamura M, Tomemori Y, Kamae K, Tanabe H, et al. The gene encoding a newly discovered protein, chorein, is mutated in chorea-acanthocytosis. Nat Genet. 2001 Jun;28(2):121–2.

17. Danek A, Rubio JP, Rampoldi L, Ho M, Dobson-Stone C, Tison F, et al. McLeod neuroacanthocytosis: genotype and phenotype. Ann Neurol. 2001 Dec;50(6):755–64.

18. Inzelberg R, Weinberger M, Gak E. Benign hereditary chorea: an update. Parkinsonism Relat Disord. 2011 Jun;17(5):301–7.

19. Diggle CP, Sukoff Rizzo SJ, Popiolek M, Hinttala R, Schülke JP, Kurian MA, et al. Biallelic Mutations in PDE10A Lead to Loss of Striatal PDE10A and a Hyperkinetic Movement Disorder with Onset in Infancy. Am J Hum Genet. 2016 Apr 7;98(4):735–43.

20. Mencacci NE, Kamsteeg EJ, Nakashima K, R’Bibo L, Lynch DS, Balint B, et al. De Novo Mutations in PDE10A Cause Childhood-Onset Chorea with Bilateral Striatal Lesions. Am J Hum Genet. 2016 Apr 7;98(4):763–71.

21. Fuki M Hisama, Jennifer Friedman, Wendy H Raskind and Thomas D Bird. ADCY5 Dyskinesia. In: GeneReviews® [Internet] [Internet]. Margaret P Adam, Jerry Feldman, Ghayda M Mirzaa, Roberta A Pagon, Stephanie E Wallace, Anne Amemiya; Available from: https://www.ncbi.nlm.nih.gov/books/NBK263441/

22. Alkufri F, Shaag A, Abu-Libdeh B, Elpeleg O. Deleterious mutation in GPR88 is associated with chorea, speech delay, and learning disabilities. Neurol Genet. 2016 Jun;2(3):e64.

23. Ousingsawat J, Talbi K, Gómez-Martín H, Koy A, Fernández-Jaén A, Tekgül H, et al. Broadening the clinical spectrum: molecular mechanisms and new phenotypes of ANO3-dystonia. Brain. 2024 Jun 3;147(6):1982–95.

24. Percetti M, Zini M, Soliveri P, Cogiamanian F, Ferrara M, Orunesu E, et al. The Clinical Spectrum of ANO3-Report of a New Family and Literature Review. Mov Disord Clin Pract. 2024 Mar;11(3):289–97.

25. Charlesworth G, Plagnol V, Holmström KM, Bras J, Sheerin UM, Preza E, et al. Mutations in ANO3 cause dominant craniocervical dystonia: ion channel implicated in pathogenesis. Am J Hum Genet. 2012 Dec 7;91(6):1041–50.

26. Masuho I, Fang M, Geng C, Zhang J, Jiang H, Özgul RK, et al. Homozygous GNAL mutation associated with familial childhood-onset generalized dystonia. Neurol Genet. 2016 Jun;2(3):e78.

27. Kumar KR, Lohmann K, Masuho I, Miyamoto R, Ferbert A, Lohnau T, et al. Mutations in GNAL: a novel cause of craniocervical dystonia. JAMA Neurol. 2014 Apr;71(4):490–4.

28. Fuchs T, Saunders-Pullman R, Masuho I, Luciano MS, Raymond D, Factor S, et al. Mutations in GNAL cause primary torsion dystonia. Nat Genet. 2013 Jan;45(1):88–92.

29. Magrinelli F, Bhatia KP, Beiraghi Toosi M, Arab F, Karimiani EG, Sedighzadeh S, et al. Childhood-Onset Choreo-Dystonia Due to a Recurrent Novel Homozygous Nonsense HPCA Variant: Case Series and Literature Review. Mov Disord Clin Pract. 2023 Jan;10(1):101–8.

30. Charlesworth G, Angelova PR, Bartolomé-Robledo F, Ryten M, Trabzuni D, Stamelou M, et al. Mutations in HPCA cause autosomal-recessive primary isolated dystonia. Am J Hum Genet. 2015 Apr 2;96(4):657–65.

31. Zech M, Boesch S, Maier EM, Borggraefe I, Vill K, Laccone F, et al. Haploinsufficiency of KMT2B, Encoding the Lysine-Specific Histone Methyltransferase 2B, Results in Early-Onset Generalized Dystonia. Am J Hum Genet. 2016 Dec 1;99(6):1377–87.

32. Meyer E, Carss KJ, Rankin J, Nichols JME, Grozeva D, Joseph AP, et al. Mutations in the histone methyltransferase gene KMT2B cause complex early-onset dystonia. Nat Genet. 2017 Feb;49(2):223–37.

33. Camargos S, Scholz S, Simón-Sánchez J, Paisán-Ruiz C, Lewis P, Hernandez D, et al. DYT16, a novel young-onset dystonia-parkinsonism disorder: identification of a segregating mutation in the stress-response protein PRKRA. Lancet Neurol. 2008 Mar;7(3):207–15.

34. Fuchs T, Gavarini S, Saunders-Pullman R, Raymond D, Ehrlich ME, Bressman SB, et al. Mutations in the THAP1 gene are responsible for DYT6 primary torsion dystonia. Nat Genet. 2009 Mar;41(3):286–8.

35. Vulinovic F, Lohmann K, Rakovic A, Capetian P, Alvarez-Fischer D, Schmidt A, et al. Unraveling cellular phenotypes of novel TorsinA/TOR1A mutations. Hum Mutat. 2014 Sep;35(9):1114–22.

36. Hettich J, Ryan SD, de Souza ON, Saraiva Macedo Timmers LF, Tsai S, Atai NA, et al. Biochemical and cellular analysis of human variants of the DYT1 dystonia protein, TorsinA/TOR1A. Hum Mutat. 2014 Sep;35(9):1101–13.

37. Opal P, Tintner R, Jankovic J, Leung J, Breakefield XO, Friedman J, et al. Intrafamilial phenotypic variability of the DYT1 dystonia: from asymptomatic TOR1A gene carrier status to dystonic storm. Mov Disord. 2002 Mar;17(2):339–45.

38. Kamm C, Fischer H, Garavaglia B, Kullmann S, Sharma M, Schrader C, et al. Susceptibility to DYT1 dystonia in European patients is modified by the D216H polymorphism. Neurology. 2008 Jun 3;70(23):2261–2.

39. Ozelius LJ, Kramer PL, de Leon D, Risch N, Bressman SB, Schuback DE, et al. Strong allelic association between the torsion dystonia gene (DYT1) andloci on chromosome 9q34 in Ashkenazi Jews. Am J Hum Genet. 1992 Mar;50(3):619–28.

40. Cai X, Chen X, Wu S, Liu W, Zhang X, Zhang D, et al. Homozygous mutation of VPS16 gene is responsible for an autosomal recessive adolescent-onset primary dystonia. Sci Rep. 2016 May 12;6:25834.

41. Pott H, Brüggemann N, Reese R, Zeuner KE, Gandor F, Gruber D, et al. Truncating VPS16 Mutations Are Rare in Early Onset Dystonia. Ann Neurol. 2021 Mar;89(3):625–6.

42. Monfrini E, Zech M, Steel D, Kurian MA, Winkelmann J, Di Fonzo A. HOPS-associated neurological disorders (HOPSANDs): linking endolysosomal dysfunction to the pathogenesis of dystonia. Brain. 2021 Oct 22;144(9):2610–5.

43. Steel D, Zech M, Zhao C, Barwick KES, Burke D, Demailly D, et al. Loss-of-Function Variants in HOPS Complex Genes VPS16 and VPS41 Cause Early Onset Dystonia Associated with Lysosomal Abnormalities. Ann Neurol. 2020 Nov;88(5):867–77.

44. Zech M, Kumar KR, Reining S, Reunert J, Tchan M, Riley LG, et al. Biallelic AOPEP Loss-of-Function Variants Cause Progressive Dystonia with Prominent Limb Involvement. Mov Disord. 2022 Jan;37(1):137–47.

45. Garavaglia B, Vallian S, Romito LM, Straccia G, Capecci M, Invernizzi F, et al. AOPEP variants as a novel cause of recessive dystonia: Generalized dystonia and dystonia-parkinsonism. Parkinsonism Relat Disord. 2022 Apr;97:52–6.

46. Rosewich H, Ohlenbusch A, Huppke P, Schlotawa L, Baethmann M, Carrilho I, et al. The expanding clinical and genetic spectrum of ATP1A3-related disorders. Neurology. 2014 Mar 18;82(11):945–55.

47. Makino S, Kaji R, Ando S, Tomizawa M, Yasuno K, Goto S, et al. Reduced neuron-specific expression of the TAF1 gene is associated with X-linked dystonia-parkinsonism. Am J Hum Genet. 2007 Mar;80(3):393–406.

48. Nolte D, Niemann S, Müller U. Specific sequence changes in multiple transcript system DYT3 are associated with X-linked dystonia parkinsonism. Proc Natl Acad Sci U S A. 2003 Sep 2;100(18):10347–52.

49. Wu-Chou YH, Yeh TH, Wang CY, Lin JJ, Huang CC, Chang HC, et al. High frequency of multiexonic deletion of the GCH1 gene in a Taiwanese cohort of dopa-response dystonia. Am J Med Genet B Neuropsychiatr Genet. 2010 Jun 5;153B(4):903–8.

50. Hirano M, Imaiso Y, Ueno S. Differential splicing of the GTP cyclohydrolase I RNA in dopa-responsive dystonia. Biochem Biophys Res Commun. 1997 May 19;234(2):316–9.

51. Ichinose H, Ohye T, Takahashi E, Seki N, Hori T, Segawa M, et al. Hereditary progressive dystonia with marked diurnal fluctuation caused by mutations in the GTP cyclohydrolase I gene. Nat Genet. 1994 Nov;8(3):236–42.

52. Ormazabal A, Serrano M, Garcia-Cazorla A, Campistol J, Artuch R, Castro de Castro P, et al. Deletion in the tyrosine hydroxylase gene in a patient with a mild phenotype. Mov Disord. 2011 Jul;26(8):1558–60.

53. Stamelou M, Mencacci NE, Cordivari C, Batla A, Wood NW, Houlden H, et al. Myoclonus-dystonia syndrome due to tyrosine hydroxylase deficiency. Neurology. 2012 Jul 31;79(5):435–41.

54. DE Lonlay P, Nassogne MC, van Gennip AH, van Cruchten AC, Billatte de Villemeur T, Cretz M, et al. Tyrosine hydroxylase deficiency unresponsive to L-dopa treatment with unusual clinical and biochemical presentation. J Inherit Metab Dis. 2000 Dec;23(8):819–25.

55. Deng IB, Follett J, Bu M, Farrer MJ. DNAJC12 in Monoamine Metabolism, Neurodevelopment, and Neurodegeneration. Mov Disord. 2024 Feb;39(2):249–58.

56. Tuschl K, Meyer E, Valdivia LE, Zhao N, Dadswell C, Abdul-Sada A, et al. Mutations in SLC39A14 disrupt manganese homeostasis and cause childhood-onset parkinsonism-dystonia. Nat Commun. 2016 May 27;7:11601.

57. Mencacci NE, Rubio-Agusti I, Zdebik A, Asmus F, Ludtmann MHR, Ryten M, et al. A missense mutation in KCTD17 causes autosomal dominant myoclonus-dystonia. Am J Hum Genet. 2015 Jun 4;96(6):938–47.

58. Krenn M, Sommer R, Sycha T, Zech M. GNAO1 Haploinsufficiency Associated with a Mild Delayed-Onset Dystonia Phenotype. Mov Disord. 2022 Dec;37(12):2464–6.

59. Lasa-Aranzasti A, Cazurro-Gutiérrez A, Bescós A, González V, Ispierto L, Tardáguila M, et al. 16q12.2q21 deletion: A newly recognized cause of dystonia related to GNAO1 haploinsufficiency. Parkinsonism Relat Disord. 2022 Oct;103:112–4.

60. Wirth T, Garone G, Kurian MA, Piton A, Millan F, Telegrafi A, et al. Highlighting the Dystonic Phenotype Related to GNAO1. Mov Disord. 2022 Jul;37(7):1547–54.

61. Ozcanyuz DG, Incecik F, Herguner OM, Mungan NO, Bozdogan ST. Dysarthria, Ataxia, and Dystonia Associated with COX20 (FAM36A) Gene Mutation: A Case Report of a Turkish Child. Ann Indian Acad Neurol. 2020;23(3):399–401.

62. Doss S, Lohmann K, Seibler P, Arns B, Klopstock T, Zühlke C, et al. Recessive dystonia-ataxia syndrome in a Turkish family caused by a COX20 (FAM36A) mutation. J Neurol. 2014 Jan;261(1):207–12.

63. Hayflick SJ, Kurian MA, Hogarth P. Neurodegeneration with brain iron accumulation. Handb Clin Neurol. 2018;147:293–305.

64. Møller LB, Horn N, Jeppesen TD, Vissing J, Wibrand F, Jennum P, et al. Clinical presentation and mutations in Danish patients with Wilson disease. Eur J Hum Genet. 2011 Sep;19(9):935–41.

65. Tuschl K, Clayton PT, Gospe SM, Gulab S, Ibrahim S, Singhi P, et al. Syndrome of hepatic cirrhosis, dystonia, polycythemia, and hypermanganesemia caused by mutations in SLC30A10, a manganese transporter in man. Am J Hum Genet. 2012 Mar 9;90(3):457–66.

66. Anagianni S, Tuschl K. Genetic Disorders of Manganese Metabolism. Curr Neurol Neurosci Rep. 2019 May 14;19(6):33.

67. Quadri M, Federico A, Zhao T, Breedveld GJ, Battisti C, Delnooz C, et al. Mutations in SLC30A10 cause parkinsonism and dystonia with hypermanganesemia, polycythemia, and chronic liver disease. Am J Hum Genet. 2012 Mar 9;90(3):467–77.

68. Indelicato E, Schlieben LD, Stenton SL, Boesch S, Skorvanek M, Necpal J, et al. Dystonia and mitochondrial disease: the movement disorder connection revisited in 900 genetically diagnosed patients. J Neurol. 2024 Jul;271(7):4685–92.

69. Pearl PL. Monoamine neurotransmitter deficiencies. Handb Clin Neurol. 2013;113:1819–25.

70. Sedel F, Saudubray JM, Roze E, Agid Y, Vidailhet M. Movement disorders and inborn errors of metabolism in adults: a diagnostic approach. J Inherit Metab Dis. 2008 Jun;31(3):308–18.

71. Kurian MA, Li Y, Zhen J, Meyer E, Hai N, Christen HJ, et al. Clinical and molecular characterisation of hereditary dopamine transporter deficiency syndrome: an observational cohort and experimental study. Lancet Neurol. 2011 Jan;10(1):54–62.

72. Marcé-Grau A, Martí-Sánchez L, Baide-Mairena H, Ortigoza-Escobar JD, Pérez-Dueñas B. Genetic defects of thiamine transport and metabolism: A review of clinical phenotypes, genetics, and functional studies. J Inherit Metab Dis. 2019 Jul;42(4):581–97.

73. Flønes I, Sztromwasser P, Haugarvoll K, Dölle C, Lykouri M, Schwarzlmüller T, et al. Novel SLC19A3 Promoter Deletion and Allelic Silencing in Biotin-Thiamine-Responsive Basal Ganglia Encephalopathy. PLoS One. 2016;11(2):e0149055.

74. Bally JF, Camargos S, Oliveira Dos Santos C, Kern DS, Lee T, Pereira da Silva-Junior F, et al. DYT-TUBB4A (DYT4 Dystonia): New Clinical and Genetic Observations. Neurology. 2021 Apr 6;96(14):e1887–97.

75. Erro R, Hersheson J, Ganos C, Mencacci NE, Stamelou M, Batla A, et al. H-ABC syndrome and DYT4: Variable expressivity or pleiotropy of TUBB4 mutations? Mov Disord. 2015 May;30(6):828–33.

76. Lenk GM, Szymanska K, Debska-Vielhaber G, Rydzanicz M, Walczak A, Bekiesinska-Figatowska M, et al. Biallelic Mutations of VAC14 in Pediatric-Onset Neurological Disease. Am J Hum Genet. 2016 Jul 7;99(1):188–94.

77. Crow YJ, Chase DS, Lowenstein Schmidt J, Szynkiewicz M, Forte GMA, Gornall HL, et al. Characterization of human disease phenotypes associated with mutations in TREX1, RNASEH2A, RNASEH2B, RNASEH2C, SAMHD1, ADAR, and IFIH1. Am J Med Genet A. 2015 Feb;167A(2):296–312.

78. Rice GI, Kasher PR, Forte GMA, Mannion NM, Greenwood SM, Szynkiewicz M, et al. Mutations in ADAR1 cause Aicardi-Goutières syndrome associated with a type I interferon signature. Nat Genet. 2012 Nov;44(11):1243–8.

79. Patrizi A, Manneschi V, Pini A, Baioni E, Ghetti P. Dyschromatosis symmetrica hereditaria associated with idiopathic torsion dystonia. A case report. Acta Derm Venereol. 1994 Mar;74(2):135–7.

80. Marcogliese PC, Shashi V, Spillmann RC, Stong N, Rosenfeld JA, Koenig MK, et al. IRF2BPL Is Associated with Neurological Phenotypes. Am J Hum Genet. 2018 Aug 2;103(2):245–60.

81. Seltzer LE, Ma M, Ahmed S, Bertrand M, Dobyns WB, Wheless J, et al. Epilepsy and outcome in FOXG1-related disorders. Epilepsia. 2014 Aug;55(8):1292–300.

82. Papa FT, Mencarelli MA, Caselli R, Katzaki E, Sampieri K, Meloni I, et al. A 3 Mb deletion in 14q12 causes severe mental retardation, mild facial dysmorphisms and Rett-like features. Am J Med Genet A. 2008 Aug 1;146A(15):1994–8.

83. Mitter D, Pringsheim M, Kaulisch M, Plümacher KS, Schröder S, Warthemann R, et al. FOXG1 syndrome: genotype-phenotype association in 83 patients with FOXG1 variants. Genet Med. 2018 Jan;20(1):98–108.

84. Fu R, Ceballos-Picot I, Torres RJ, Larovere LE, Yamada Y, Nguyen KV, et al. Genotype-phenotype correlations in neurogenetics: Lesch-Nyhan disease as a model disorder. Brain. 2014 May;137(Pt 5):1282–303.

85. Madeo A, Di Rocco M, Brassier A, Bahi-Buisson N, De Lonlay P, Ceballos-Picot I. Clinical, biochemical and genetic characteristics of a cohort of 101 French and Italian patients with HPRT deficiency. Mol Genet Metab. 2019 Jun;127(2):147–57.

86. Stenson PD, Mort M, Ball EV, Chapman M, Evans K, Azevedo L, et al. The Human Gene Mutation Database (HGMD®): optimizing its use in a clinical diagnostic or research setting. Hum Genet. 2020 Oct;139(10):1197–207.

87. Muto V, Flex E, Kupchinsky Z, Primiano G, Galehdari H, Dehghani M, et al. Biallelic SQSTM1 mutations in early-onset, variably progressive neurodegeneration. Neurology. 2018 Jul 24;91(4):e319–30.

88. Cacciagli P, Sutera-Sardo J, Borges-Correia A, Roux JC, Dorboz I, Desvignes JP, et al. Mutations in BCAP31 cause a severe X-linked phenotype with deafness, dystonia, and central hypomyelination and disorganize the Golgi apparatus. Am J Hum Genet. 2013 Sep 5;93(3):579–86.

89. Riedhammer KM, Leszinski GS, Andres S, Strobl-Wildemann G, Wagner M. First replication that biallelic variants in FITM2 cause a complex deafness-dystonia syndrome. Mov Disord. 2018 Oct;33(10):1665–6.

90. Figueroa KP, Coon H, Santos N, Velazquez L, Mederos LA, Pulst SM. Genetic analysis of age at onset variation in spinocerebellar ataxia type 2. Neurol Genet. 2017 Jun;3(3):e155.

91. Dor T, Cinnamon Y, Raymond L, Shaag A, Bouslam N, Bouhouche A, et al. KIF1C mutations in two families with hereditary spastic paraparesis and cerebellar dysfunction. J Med Genet. 2014 Feb;51(2):137–42.

92. Edvardson S, Cinnamon Y, Ta-Shma A, Shaag A, Yim YI, Zenvirt S, et al. A deleterious mutation in DNAJC6 encoding the neuronal-specific clathrin-uncoating co-chaperone auxilin, is associated with juvenile parkinsonism. PLoS One. 2012;7(5):e36458.

93. Vauthier V, Jaillard S, Journel H, Dubourg C, Jockers R, Dam J. Homozygous deletion of an 80 kb region comprising part of DNAJC6 and LEPR genes on chromosome 1P31.3 is associated with early onset obesity, mental retardation and epilepsy. Mol Genet Metab. 2012 Jul;106(3):345–50.

94. Harrer P, Schalk A, Shimura M, Baer S, Calmels N, Spitz MA, et al. Recessive NUP54 Variants Underlie Early-Onset Dystonia with Striatal Lesions. Ann Neurol. 2023 Feb;93(2):330–5.

95. Basel-Vanagaite L, Muncher L, Straussberg R, Pasmanik-Chor M, Yahav M, Rainshtein L, et al. Mutated nup62 causes autosomal recessive infantile bilateral striatal necrosis. Ann Neurol. 2006 Aug;60(2):214–22.

96. Reid KM, Steel D, Nair S, Bhate S, Biassoni L, Sudhakar S, et al. Loss-of-Function Variants in DRD1 in Infantile Parkinsonism-Dystonia. Cells. 2023 Mar 30;12(7):1046.

97. van der Weijden MCM, Rodriguez-Contreras D, Delnooz CCS, Robinson BG, Condon AF, Kielhold ML, et al. A Gain-of-Function Variant in Dopamine D2 Receptor and Progressive Chorea and Dystonia Phenotype. Mov Disord. 2021 Mar;36(3):729–39.

98. Nasca A, Mencacci NE, Invernizzi F, Zech M, Keller Sarmiento IJ, Legati A, et al. Variants in ATP5F1B are associated with dominantly inherited dystonia. Brain. 2023 Jul 3;146(7):2730–8.

99. Perlman S. Hereditary Ataxia Overview. In: Adam MP, Feldman J, Mirzaa GM, Pagon RA, Wallace SE, Bean LJ, et al., editors. GeneReviews® [Internet]. Seattle (WA): University of Washington, Seattle; 1993 [cited 2024 Sep 7]. Available from: http://www.ncbi.nlm.nih.gov/books/NBK1138/

100. Cui ZT, Mao ZT, Yang R, Li JJ, Jia SS, Zhao JL, et al. Spinocerebellar ataxias: from pathogenesis to recent therapeutic advances. Front Neurosci. 2024;18:1422442.

101. Liu G, Bissler JJ, Sinden RR, Leffak M. Unstable spinocerebellar ataxia type 10 (ATTCT*(AGAAT) repeats are associated with aberrant replication at the ATX10 locus and replication origin-dependent expansion at an ectopic site in human cells. Mol Cell Biol. 2007 Nov;27(22):7828–38.

102. Toyoshima Y, Onodera O, Yamada M, Tsuji S, Takahashi H. Spinocerebellar Ataxia Type 17. In: Adam MP, Feldman J, Mirzaa GM, Pagon RA, Wallace SE, Bean LJ, et al., editors. GeneReviews® [Internet]. Seattle (WA): University of Washington, Seattle; 1993 [cited 2024 Sep 7]. Available from: http://www.ncbi.nlm.nih.gov/books/NBK1438/

103. Kobayashi H, Abe K, Matsuura T, Ikeda Y, Hitomi T, Akechi Y, et al. Expansion of intronic GGCCTG hexanucleotide repeat in NOP56 causes SCA36, a type of spinocerebellar ataxia accompanied by motor neuron involvement. Am J Hum Genet. 2011 Jul 15;89(1):121–30.

104. Ishikawa K. Spinocerebellar ataxia type 31 (SCA31). J Hum Genet. 2023 Mar;68(3):153–6.

105. Casey HL, Gomez CM. Spinocerebellar Ataxia Type 6. In: Adam MP, Feldman J, Mirzaa GM, Pagon RA, Wallace SE, Bean LJ, et al., editors. GeneReviews® [Internet]. Seattle (WA): University of Washington, Seattle; 1993 [cited 2024 Sep 7]. Available from: http://www.ncbi.nlm.nih.gov/books/NBK1140/

106. Olszewska DA, Shetty A, Rajalingam R, Rodriguez-Antiguedad J, Hamed M, Huang J, et al. Genotype-phenotype relations for episodic ataxia genes: MDSGene systematic review. Eur J Neurol. 2023 Oct;30(10):3377–93.

107. Cortese A, Tozza S, Yau WY, Rossi S, Beecroft SJ, Jaunmuktane Z, et al. Cerebellar ataxia, neuropathy, vestibular areflexia syndrome due to RFC1 repeat expansion. Brain. 2020 Feb 1;143(2):480–90.

108. Pellerin D, Danzi MC, Wilke C, Renaud M, Fazal S, Dicaire MJ, et al. Deep Intronic FGF14 GAA Repeat Expansion in Late-Onset Cerebellar Ataxia. N Engl J Med. 2023 Jan 12;388(2):128–41.

109. Rafehi H, Read J, Szmulewicz DJ, Davies KC, Snell P, Fearnley LG, et al. An intronic GAA repeat expansion in FGF14 causes the autosomal-dominant adult-onset ataxia SCA27B/ATX-FGF14. Am J Hum Genet. 2023 Jun 1;110(6):1018.

110. Prades S, Melo de Gusmao C, Grimaldi S, Shiloh-Malawsky Y, Felton T, Houlden H. DRPLA. In: Adam MP, Feldman J, Mirzaa GM, Pagon RA, Wallace SE, Amemiya A, editors. GeneReviews® [Internet]. Seattle (WA): University of Washington, Seattle; 1993 [cited 2024 Oct 26]. Available from: http://www.ncbi.nlm.nih.gov/books/NBK1491/

111. Chaudhry A, Athanasiou-Fragkouli A, Garcia-Moreno H, Abadias SP, Greenfield J, Shiloh-Malawsky Y, et al. Correction to: DRPLA: understanding the natural history and developing biomarkers to accelerate therapeutic trials in a globally rare repeat expansion disorder. J Neurol. 2021 Aug;268(8):3042.

112. Hunter JE, Berry-Kravis E, Hipp H, Todd PK. FMR1 Disorders. In: Adam MP, Feldman J, Mirzaa GM, Pagon RA, Wallace SE, Bean LJ, et al., editors. GeneReviews® [Internet]. Seattle (WA): University of Washington, Seattle; 1993 [cited 2024 Sep 7]. Available from: http://www.ncbi.nlm.nih.gov/books/NBK1384/

113. Zeviani M, Simonati A, Bindoff LA. Ataxia in mitochondrial disorders. Handb Clin Neurol. 2012;103:359–72.

114. Kara B, Uzümcü A, Uyguner O, Rosti RO, Koçbaş A, Ozmen M, et al. Ataxia with vitamin E deficiency associated with deafness. Turk J Pediatr. 2008;50(5):471–5.

115. Schuelke M. Ataxia with Vitamin E Deficiency. In: Adam MP, Feldman J, Mirzaa GM, Pagon RA, Wallace SE, Bean LJ, et al., editors. GeneReviews® [Internet]. Seattle (WA): University of Washington, Seattle; 1993 [cited 2024 Sep 7]. Available from: http://www.ncbi.nlm.nih.gov/books/NBK1241/

116. Gurram S, Holla VV, Sriram N, Phulpagar P, Jha S, Sharma P, et al. A Rare Case of Ophthalmoplegia with Ataxia in Genetically Proven Abetalipoproteinemia. Mov Disord Clin Pract. 2023 Mar;10(3):514–7.

117. Hoffman-Zacharska D, Mazurczak T, Zajkowski T, Tataj R, Górka-Skoczylas P, Połatyńska K, et al. Friedreich ataxia is not only a GAA repeats expansion disorder: implications for molecular testing and counselling. J Appl Genet. 2016 Aug;57(3):349–55.

118. Anheim M, Mariani LL, Calvas P, Cheuret E, Zagnoli F, Odent S, et al. Exonic deletions of FXN and early-onset Friedreich ataxia. Arch Neurol. 2012 Jul;69(7):912–6.

119. Zühlke CH, Dalski A, Habeck M, Straube K, Hedrich K, Hoeltzenbein M, et al. Extension of the mutation spectrum in Friedreich's ataxia: detection of an exon deletion and novel missense mutations. Eur J Hum Genet. 2004 Nov;12(11):979–82.

120. Bidichandani SI, Delatycki MB. Friedreich Ataxia. In: Adam MP, Feldman J, Mirzaa GM, Pagon RA, Wallace SE, Bean LJ, et al., editors. GeneReviews® [Internet]. Seattle (WA): University of Washington, Seattle; 1993 [cited 2024 Sep 7]. Available from: http://www.ncbi.nlm.nih.gov/books/NBK1281/

121. Schon K, van Os NJH, Oscroft N, Baxendale H, Scoffings D, Ray J, et al. Genotype, extrapyramidal features, and severity of variant ataxia-telangiectasia. Ann Neurol. 2019 Feb;85(2):170–80.

122. Arning L, Schöls L, Cin H, Souquet M, Epplen JT, Timmann D. Identification and characterisation of a large senataxin (SETX) gene duplication in ataxia with ocular apraxia type 2 (AOA2). Neurogenetics. 2008 Oct;9(4):295–9.

123. Anheim M, Monga B, Fleury M, Charles P, Barbot C, Salih M, et al. Ataxia with oculomotor apraxia type 2: clinical, biological and genotype/phenotype correlation study of a cohort of 90 patients. Brain. 2009 Oct;132(Pt 10):2688–98.

124. Bernard V, Minnerop M, Bürk K, Kreuz F, Gillessen-Kaesbach G, Zühlke C. Exon deletions and intragenic insertions are not rare in ataxia with oculomotor apraxia 2. BMC Med Genet. 2009 Sep 11;10:87.

125. McConville CM, Stankovic T, Byrd PJ, McGuire GM, Yao QY, Lennox GG, et al. Mutations associated with variant phenotypes in ataxia-telangiectasia. Am J Hum Genet. 1996 Aug;59(2):320–30.

126. Takiyama Y. Sacsinopathies: sacsin-related ataxia. Cerebellum. 2007;6(4):353–9.

127. Renaud M, Anheim M, Kamsteeg EJ, Mallaret M, Mochel F, Vermeer S, et al. Autosomal recessive cerebellar ataxia type 3 due to ANO10 mutations: delineation and genotype-phenotype correlation study. JAMA Neurol. 2014 Oct;71(10):1305–10.

128. Milovanović A, Westenberger A, Stanković I, Tamaš O, Branković M, Marjanović A, et al. ANO10-Related Spinocerebellar Ataxia: MDSGene Systematic Literature Review and a Romani Case Series. Mov Disord. 2024 May;39(5):887–92.

129. Dupré N, Gros-Louis F, Chrestian N, Verreault S, Brunet D, de Verteuil D, et al. Clinical and genetic study of autosomal recessive cerebellar ataxia type 1. Ann Neurol. 2007 Jul;62(1):93–8.

130. Mademan I, Harmuth F, Giordano I, Timmann D, Magri S, Deconinck T, et al. Multisystemic SYNE1 ataxia: confirming the high frequency and extending the mutational and phenotypic spectrum. Brain. 2016 Aug;139(Pt 8):e46.

131. Synofzik M, Smets K, Mallaret M, Di Bella D, Gallenmüller C, Baets J, et al. SYNE1 ataxia is a common recessive ataxia with major non-cerebellar features: a large multi-centre study. Brain. 2016 May;139(Pt 5):1378–93.

132. Baumann M, Steichen-Gersdorf E, Krabichler B, Petersen BS, Weber U, Schmidt WM, et al. Homozygous SYNE1 mutation causes congenital onset of muscular weakness with distal arthrogryposis: a genotype-phenotype correlation. Eur J Hum Genet. 2017 Feb;25(2):262–6.

133. Marelli C, van de Leemput J, Johnson JO, Tison F, Thauvin-Robinet C, Picard F, et al. SCA15 due to large ITPR1 deletions in a cohort of 333 white families with dominant ataxia. Arch Neurol. 2011 May;68(5):637–43.

134. Zambonin JL, Bellomo A, Ben-Pazi H, Everman DB, Frazer LM, Geraghty MT, et al. Spinocerebellar ataxia type 29 due to mutations in ITPR1: a case series and review of this emerging congenital ataxia. Orphanet J Rare Dis. 2017 Jun 28;12(1):121.

135. Duarri A, Jezierska J, Fokkens M, Meijer M, Schelhaas HJ, den Dunnen WFA, et al. Mutations in potassium channel kcnd3 cause spinocerebellar ataxia type 19. Ann Neurol. 2012 Dec;72(6):870–80.

136. Li M, Liu F, Hao X, Fan Y, Li J, Hu Z, et al. Rare KCND3 Loss-of-Function Mutation Associated With the SCA19/22. Front Mol Neurosci. 2022;15:919199.

137. Romaniello R, Citterio A, Panzeri E, Arrigoni F, De Rinaldis M, Trabacca A, et al. Novel SPTBN2 gene mutation and first intragenic deletion in early onset spinocerebellar ataxia type 5. Ann Clin Transl Neurol. 2021 Apr;8(4):956–63.

138. Yıldız Bölükbaşı E, Afzal M, Mumtaz S, Ahmad N, Malik S, Tolun A. Progressive SCAR14 with unclear speech, developmental delay, tremor, and behavioral problems caused by a homozygous deletion of the SPTBN2 pleckstrin homology domain. Am J Med Genet A. 2017 Sep;173(9):2494–9.

139. Smets K, Deconinck T, Baets J, Sieben A, Martin JJ, Smouts I, et al. Partial deletion of AFG3L2 causing spinocerebellar ataxia type 28. Neurology. 2014 Jun 10;82(23):2092–100.

140. Roux T, Barbier M, Papin M, Davoine CS, Sayah S, Coarelli G, et al. Clinical, neuropathological, and genetic characterization of STUB1 variants in cerebellar ataxias: a frequent cause of predominant cognitive impairment. Genet Med. 2020 Nov;22(11):1851–62.

141. Genis D, Ortega-Cubero S, San Nicolás H, Corral J, Gardenyes J, de Jorge L, et al. Heterozygous STUB1 mutation causes familial ataxia with cognitive affective syndrome (SCA48). Neurology. 2018 Nov 20;91(21):e1988–98.

142. Sanchez-Flores M, Corral-Juan M, Gasch-Navalón E, Cirillo D, Sanchez I, Matilla-Dueñas A. Novel genotype-phenotype correlations, differential cerebellar allele-specific methylation, and a common origin of the (ATTTC)n insertion in spinocerebellar ataxia type 37. Hum Genet. 2024 Mar;143(3):211–32.

143. Hajjari M, Tahmasebi-Birgani M, Mohammadi-Asl J, Nasiri H, Kollaee A, Mahmoodi M, et al. Exome sequencing found a novel homozygous deletion in ADCK3 gene involved in autosomal recessive spinocerebellar ataxia. Gene. 2019 Aug 5;708:10–3.

144. Corbett MA, Depienne C, Veneziano L, Klein KM, Brancati F, Guerrini R, et al. Genetics of familial adult myoclonus epilepsy: From linkage studies to noncoding repeat expansions. Epilepsia. 2023 Jun;64 Suppl 1(Suppl 1):S14–21.

145. Pennacchio LA, Lehesjoki AE, Stone NE, Willour VL, Virtaneva K, Miao J, et al. Mutations in the gene encoding cystatin B in progressive myoclonus epilepsy (EPM1). Science. 1996 Mar 22;271(5256):1731–4.

146. Assenza G, Benvenga A, Gennaro E, Tombini M, Campana C, Assenza F, et al. A novel c132-134del mutation in Unverricht-Lundborg disease and the review of literature of heterozygous compound patients. Epilepsia. 2017 Feb;58(2):e31–5.

147. Koskenkorva P, Hyppönen J, Aikiä M, Mervaala E, Kiviranta T, Eriksson K, et al. Severer phenotype in Unverricht-Lundborg disease (EPM1) patients compound heterozygous for the dodecamer repeat expansion and the c.202C>T mutation in the CSTB gene. Neurodegener Dis. 2011;8(6):515–22.

148. Canafoglia L, Gennaro E, Capovilla G, Gobbi G, Boni A, Beccaria F, et al. Electroclinical presentation and genotype-phenotype relationships in patients with Unverricht-Lundborg disease carrying compound heterozygous CSTB point and indel mutations. Epilepsia. 2012 Dec;53(12):2120–7.

149. Jansen AC, Andermann E. Progressive Myoclonus Epilepsy, Lafora Type. In: Adam MP, Feldman J, Mirzaa GM, Pagon RA, Wallace SE, Bean LJ, et al., editors. GeneReviews® [Internet]. Seattle (WA): University of Washington, Seattle; 1993 [cited 2024 Sep 7]. Available from: http://www.ncbi.nlm.nih.gov/books/NBK1389/

150. Turnbull J, Tiberia E, Striano P, Genton P, Carpenter S, Ackerley CA, et al. Lafora disease. Epileptic Disord. 2016 Sep 1;18(S2):38–62.

151. Velez-Bartolomei F, Lee C, Enns G. MERRF. In: Adam MP, Feldman J, Mirzaa GM, Pagon RA, Wallace SE, Bean LJ, et al., editors. GeneReviews® [Internet]. Seattle (WA): University of Washington, Seattle; 1993 [cited 2024 Sep 7]. Available from: http://www.ncbi.nlm.nih.gov/books/NBK1520/

152. Ghaoui R, Sue CM. Movement disorders in mitochondrial disease. J Neurol. 2018 May;265(5):1230–40.

153. Prades S, Melo de Gusmao C, Grimaldi S, Shiloh-Malawsky Y, Felton T, Houlden H. DRPLA. In: Adam MP, Feldman J, Mirzaa GM, Pagon RA, Wallace SE, Amemiya A, editors. GeneReviews® [Internet]. Seattle (WA): University of Washington, Seattle; 1993 [cited 2024 Oct 26]. Available from: http://www.ncbi.nlm.nih.gov/books/NBK1491/

154. Canafoglia L, Robbiano A, Pareyson D, Panzica F, Nanetti L, Giovagnoli AR, et al. Expanding sialidosis spectrum by genome-wide screening: NEU1 mutations in adult-onset myoclonus. Neurology. 2014 Jun 3;82(22):2003–6.

155. Park JK, Orvisky E, Tayebi N, Kaneski C, Lamarca ME, Stubblefield BK, et al. Myoclonic epilepsy in Gaucher disease: genotype-phenotype insights from a rare patient subgroup. Pediatr Res. 2003 Mar;53(3):387–95.

156. Atasu B, Acarlı ANO, Bilgic B, Baykan B, Demir E, Ozluk Y, et al. Genotype-Phenotype correlations of SCARB2 associated clinical presentation: a case report and in-depth literature review. BMC Neurol. 2022 Mar 28;22(1):122.

157. Xiao C, Ahn H, Kibrom S, Toro C. SCARB2-Related Action Myoclonus – Renal Failure Syndrome. In: Adam MP, Feldman J, Mirzaa GM, Pagon RA, Wallace SE, Bean LJ, et al., editors. GeneReviews® [Internet]. Seattle (WA): University of Washington, Seattle; 1993 [cited 2024 Sep 7]. Available from: http://www.ncbi.nlm.nih.gov/books/NBK333437/

158. Tao H, Manak JR, Sowers L, Mei X, Kiyonari H, Abe T, et al. Mutations in prickle orthologs cause seizures in flies, mice, and humans. Am J Hum Genet. 2011 Feb 11;88(2):138–49.

159. Bosoi CM, Capra V, Allache R, Trinh VQH, De Marco P, Merello E, et al. Identification and characterization of novel rare mutations in the planar cell polarity gene PRICKLE1 in human neural tube defects. Hum Mutat. 2011 Dec;32(12):1371–5.

160. Bassuk AG, Sherr EH. A de novo mutation in PRICKLE1 in fetal agenesis of the corpus callosum and polymicrogyria. J Neurogenet. 2015;29(4):174–7.

161. Todd BP, Bassuk AG. A de novo mutation in PRICKLE1 associated with myoclonic epilepsy and autism spectrum disorder. J Neurogenet. 2018 Dec;32(4):313–5.

162. Algahtani H, Al-Hakami F, Al-Shehri M, Shirah B, Al-Qahtani MH, Abdulkareem AA, et al. A very rare form of autosomal dominant progressive myoclonus epilepsy caused by a novel variant in the PRICKLE1 gene. Seizure. 2019 Jul;69:133–9.

163. Corbett MA, Schwake M, Bahlo M, Dibbens LM, Lin M, Gandolfo LC, et al. A mutation in the Golgi Qb-SNARE gene GOSR2 causes progressive myoclonus epilepsy with early ataxia. Am J Hum Genet. 2011 May 13;88(5):657–63.

164. Zhou J, Tawk M, Tiziano FD, Veillet J, Bayes M, Nolent F, et al. Spinal muscular atrophy associated with progressive myoclonic epilepsy is caused by mutations in ASAH1. Am J Hum Genet. 2012 Jul 13;91(1):5–14.

165. Williams LJ, Waller S, Qiu J, Innes E, Elserafy N, Procopis P, et al. DHDDS and NUS1: A Converging Pathway and Common Phenotype. Mov Disord Clin Pract. 2024 Jan;11(1):76–85.

166. Riboldi GM, Monfrini E, Stahl C, Frucht SJ. NUS1 and Epilepsy-myoclonus-ataxia Syndrome: An Under-recognized Entity? Tremor Other Hyperkinet Mov (N Y). 2022;12:21.

167. Monfrini E, Miller C, Frucht SJ, Di Fonzo A, Riboldi GM. Progressive myoclonus without epilepsy due to a NUS1 frameshift insertion: Dyssynergia cerebellaris myoclonica revisited. Parkinsonism Relat Disord. 2022 May;98:53–5.

168. Grünewald A, Djarmati A, Lohmann-Hedrich K, Farrell K, Zeller JA, Allert N, et al. Myoclonus-dystonia: significance of large SGCE deletions. Hum Mutat. 2008 Feb;29(2):331–2.

169. Asmus F, Hjermind LE, Dupont E, Wagenstaller J, Haberlandt E, Munz M, et al. Genomic deletion size at the epsilon-sarcoglycan locus determines the clinical phenotype. Brain. 2007 Oct;130(Pt 10):2736–45.

170. DeBerardinis RJ, Conforto D, Russell K, Kaplan J, Kollros PR, Zackai EH, et al. Myoclonus in a patient with a deletion of the epsilon-sarcoglycan locus on chromosome 7q21. Am J Med Genet A. 2003 Aug 15;121A(1):31–6.

171. Roze E, Apartis E, Clot F, Dorison N, Thobois S, Guyant-Marechal L, et al. Myoclonus-dystonia: clinical and electrophysiologic pattern related to SGCE mutations. Neurology. 2008 Mar 25;70(13):1010–6.

172. Raymond D, Saunders-Pullman R, de Carvalho Aguiar P, Schule B, Kock N, Friedman J, et al. Phenotypic spectrum and sex effects in eleven myoclonus-dystonia families with epsilon-sarcoglycan mutations. Mov Disord. 2008 Mar 15;23(4):588–92.

173. Tezenas du Montcel S, Clot F, Vidailhet M, Roze E, Damier P, Jedynak CP, et al. Epsilon sarcoglycan mutations and phenotype in French patients with myoclonic syndromes. J Med Genet. 2006 May;43(5):394–400.

174. Raymond D, Saunders-Pullman R, Ozelius L. SGCE Myoclonus-Dystonia. In: Adam MP, Feldman J, Mirzaa GM, Pagon RA, Wallace SE, Bean LJ, et al., editors. GeneReviews® [Internet]. Seattle (WA): University of Washington, Seattle; 1993 [cited 2024 Sep 7]. Available from: http://www.ncbi.nlm.nih.gov/books/NBK1414/

175. Williams L, Waller SE, Bradley M, Lockhart A, Narayanan RK, Kumar KR, et al. ATP1A3 related disease manifesting as rapid onset dystonia-parkinsonism with prominent myoclonus and exaggerated startle. Parkinsonism Relat Disord. 2023 Dec;117:105864.

176. Semaka A, Hayden MR. Evidence-based genetic counselling implications for Huntington disease intermediate allele predictive test results. Clin Genet. 2014 Apr;85(4):303–11.

177. Caron NS, Wright GE, Hayden MR. Huntington Disease. In: Adam MP, Feldman J, Mirzaa GM, Pagon RA, Wallace SE, Bean LJ, et al., editors. GeneReviews® [Internet]. Seattle (WA): University of Washington, Seattle; 1993 [cited 2024 Sep 7]. Available from: http://www.ncbi.nlm.nih.gov/books/NBK1305/

178. Hall PL, Laine R, Alexander JJ, Ankala A, Teot LA, Lidov HGW, et al. GM2 Activator Deficiency Caused by a Homozygous Exon 2 Deletion in GM2A. JIMD Rep. 2018;38:61–5.

179. Nie S, Chen G, Cao X, Zhang Y. Cerebrotendinous xanthomatosis: a comprehensive review of pathogenesis, clinical manifestations, diagnosis, and management. Orphanet J Rare Dis. 2014 Nov 26;9:179.

180. Pretegiani E, Rosini F, Federighi P, Cerase A, Dotti MT, Rufa A. Pendular nystagmus, palatal tremor and progressive ataxia in GM2-gangliosidosis. Eur J Neurol. 2015 Jun;22(6):e67-69.

181. Knight MA, Gardner RJM, Bahlo M, Matsuura T, Dixon JA, Forrest SM, et al. Dominantly inherited ataxia and dysphonia with dentate calcification: spinocerebellar ataxia type 20. Brain. 2004 May;127(Pt 5):1172–81.

182. Samuel M, Torun N, Tuite PJ, Sharpe JA, Lang AE. Progressive ataxia and palatal tremor (PAPT): clinical and MRI assessment with review of palatal tremors. Brain. 2004 Jun;127(Pt 6):1252–68.

183. Nicastro N, Ranza E, Antonarakis SE, Horvath J. Pure Progressive Ataxia and Palatal Tremor (PAPT) Associated with a New Polymerase Gamma (POLG) Mutation. Cerebellum. 2016 Dec;15(6):829–31.

184. Peters L, Depienne C, Klebe S. Familial adult myoclonic epilepsy (FAME): clinical features, molecular characteristics, pathophysiological aspects and diagnostic work-up. Med Genet. 2021 Dec;33(4):311–8.

185. van Rootselaar AF, van den Maagdenberg AMJM, Depienne C, Tijssen MAJ. Pentameric repeat expansions: cortical myoclonus or cortical tremor? Brain. 2020 Oct 1;143(10):e86.

186. Scheffer IE, Zuberi S, Mefford HC, Guerrini R, McTague A. Developmental and epileptic encephalopathies. Nat Rev Dis Primers. 2024 Sep 5;10(1):61.

187. Ebrahimi-Fakhari D, Saffari A, Westenberger A, Klein C. The evolving spectrum of PRRT2-associated paroxysmal diseases. Brain. 2015 Dec;138(Pt 12):3476–95.

188. Erro R, Sheerin UM, Bhatia KP. Paroxysmal dyskinesias revisited: a review of 500 genetically proven cases and a new classification. Mov Disord. 2014 Aug;29(9):1108–16.

189. Leen WG, Klepper J, Verbeek MM, Leferink M, Hofste T, van Engelen BG, et al. Glucose transporter-1 deficiency syndrome: the expanding clinical and genetic spectrum of a treatable disorder. Brain. 2010 Mar;133(Pt 3):655–70.

190. Yang H, Wang D, Engelstad K, Bagay L, Wei Y, Rotstein M, et al. Glut1 deficiency syndrome and erythrocyte glucose uptake assay. Ann Neurol. 2011 Dec;70(6):996–1005.

191. Graves TD, Cha YH, Hahn AF, Barohn R, Salajegheh MK, Griggs RC, et al. Episodic ataxia type 1: clinical characterization, quality of life and genotype-phenotype correlation. Brain. 2014 Apr;137(Pt 4):1009–18.

192. Riant F, Mourtada R, Saugier-Veber P, Tournier-Lasserve E. Large CACNA1A deletion in a family with episodic ataxia type 2. Arch Neurol. 2008 Jun;65(6):817–20.

193. Ishikawa K, Tanaka H, Saito M, Ohkoshi N, Fujita T, Yoshizawa K, et al. Japanese families with autosomal dominant pure cerebellar ataxia map to chromosome 19p13.1-p13.2 and are strongly associated with mild CAG expansions in the spinocerebellar ataxia type 6 gene in chromosome 19p13.1. Am J Hum Genet. 1997 Aug;61(2):336–46.

194. Hassan A. Episodic Ataxias: Primary and Secondary Etiologies, Treatment, and Classification Approaches. Tremor Other Hyperkinet Mov (N Y). 2023;13:9.

195. Escayg A, De Waard M, Lee DD, Bichet D, Wolf P, Mayer T, et al. Coding and noncoding variation of the human calcium-channel beta4-subunit gene CACNB4 in patients with idiopathic generalized epilepsy and episodic ataxia. Am J Hum Genet. 2000 May;66(5):1531–9.

196. Conroy J, McGettigan P, Murphy R, Webb D, Murphy SM, McCoy B, et al. A novel locus for episodic ataxia:UBR4 the likely candidate. Eur J Hum Genet. 2014 Apr;22(4):505–10.

197. Piarroux J, Riant F, Humbertclaude V, Remerand G, Hadjadj J, Rejou F, et al. FGF14-related episodic ataxia: delineating the phenotype of Episodic Ataxia type 9. Ann Clin Transl Neurol. 2020 Apr;7(4):565–72.

198. Gazulla J, Izquierdo-Alvarez S, Ruiz-Fernández E, Lázaro-Romero A, Berciano J. Episodic Vestibulocerebellar Ataxia Associated with a CACNA1G Missense Variant. Case Rep Neurol. 2021;13(2):347–54.
